# Longitudinal models for the progression of disease portfolios in a nationwide chronic heart disease population

**DOI:** 10.1371/journal.pone.0284496

**Published:** 2023-04-20

**Authors:** Nikolaj Normann Holm, Anne Frølich, Ove Andersen, Helle Gybel Juul-Larsen, Anders Stockmarr

**Affiliations:** 1 Department of Applied Mathematics and Computer Science, Technical University of Denmark, Kgs. Lyngby, Denmark; 2 Innovation and Research Centre for Multimorbidity, Slagelse Hospital, Slagelse, Denmark; 3 Department of Public Health, University of Copenhagen, Copenhagen, Denmark; 4 Department of Clinical Research, Copenhagen University Hospital Amager and Hvidovre, Hvidovre, Denmark; 5 Department of Clinical Medicine, University of Copenhagen, Copenhagen, Denmark; 6 Emergency Department, Copenhagen University Hospital Amager and Hvidovre, Hvidovre, Denmark; Kyung Hee University School of Medicine, KOREA, REPUBLIC OF

## Abstract

**Background and aim:**

With multimorbidity becoming increasingly prevalent in the ageing population, addressing the epidemiology and development of multimorbidity at a population level is needed. Individuals subject to chronic heart disease are widely multimorbid, and population-wide longitudinal studies on their chronic disease trajectories are few.

**Methods:**

Disease trajectory networks of expected disease portfolio development and chronic condition prevalences were used to map sex and socioeconomic multimorbidity patterns among chronic heart disease patients. Our data source was all Danish individuals aged 18 years and older at some point in 1995-2015, consisting of 6,048,700 individuals. We used algorithmic diagnoses to obtain chronic disease diagnoses and included individuals who received a heart disease diagnosis. We utilized a general Markov framework considering combinations of chronic diagnoses as multimorbidity states. We analyzed the time until a possible new diagnosis, termed the diagnosis postponement time, in addition to transitions to new diagnoses. We modelled the postponement times by exponential models and transition probabilities by logistic regression models.

**Findings:**

Among the cohort of 766,596 chronic heart disease diagnosed individuals, the prevalence of multimorbidity was 84.36% and 88.47% for males and females, respectively. We found sex-related differences within the chronic heart disease trajectories. Female trajectories were dominated by osteoporosis and male trajectories by cancer. We found sex important in developing most conditions, especially osteoporosis, chronic obstructive pulmonary disease and diabetes. A socioeconomic gradient was observed where diagnosis postponement time increases with educational attainment. Contrasts in disease portfolio development based on educational attainment were found for both sexes, with chronic obstructive pulmonary disease and diabetes more prevalent at lower education levels, compared to higher.

**Conclusions:**

Disease trajectories of chronic heart disease diagnosed individuals are heavily complicated by multimorbidity. Therefore, it is essential to consider and study chronic heart disease, taking into account the individuals’ entire disease portfolio.

## Introduction

Multimorbidity, defined as the coexistence of two or more chronic conditions within the same individual [1], is increasingly prevalent, primarily due to the ageing population and improved medical technologies [2–4]. The treatment of a specific diagnosis becomes complicated for multimorbid individuals due to the presence of additional conditions, posing two distinct but related challenges: the high disease burden [5] and the high treatment burden containing several outpatient visits and participation in, e.g. rehabilitation programs [6]. Not surprisingly, the treatment burden is similarly reflected in the high prevalence of polypharmacy within multimorbid individuals, especially those of older age [7, 8]. This constitutes a challenge for healthcare systems, as individuals subject to multimorbidity utilize healthcare resources substantially more than healthy individuals or individuals with a single disease [9, 10]. The disease burden of the multimorbid is often reflected in lower quality of life [11] and higher mortality rates [12]. Individuals diagnosed with chronic heart disease (HD) are of particular interest, as cardiovascular diseases are associated with a wide variety of additional conditions, spanning both somatic and psychiatric chronic diseases and further is a common condition [13–17].

In order to address the epidemiology and consequences of multimorbidity at a population level, studies based on population-wide high-quality data such as national registries are needed. Population-wide analysis of electronic health records (EHR) on multimorbidity has previously revealed specific prevalent disease combinations. The authors of [18] examined the prevalence related to combinations of groups of diagnoses of size two, three, four and five in the adult Danish population. The combination of the cardiovascular and musculoskeletal diagnosis groups was the most frequent. Additionally, among the top five most prevalent combinations of diagnoses of sizes 2–5, the cardiovascular or musculoskeletal groups were present. Cardiovascular and musculoskeletal diagnoses were similarly present in a large multimorbidity cluster identified in [19], a cluster analysis based on chronic conditions of middle-aged and older adults in the UK. This cluster also included respiratory and neurodegenerative diseases. Some of the revealed multimorbidity patterns contain known risk factor associations (e.g. chronic obstructive pulmonary disease (COPD) for cardiovascular diseases [20]).

Only a few EHR studies have considered longitudinal disease trajectories [21–23]. In a discovery-driven temporal analysis of ICD-10 disease trajectories in the entire Danish population [21], the authors found a temporal cardiovascular disease cluster. Furthermore, the musculoskeletal condition gout was discovered to be central to the cardiovascular cluster. Cardiovascular and musculoskeletal diseases were similarly prevalent in a separate COPD cluster showing a progression from cardiovascular conditions towards COPD. In the study, disease trajectories were constructed from pairs of diagnoses by considering the order of diagnosis codes from the secondary healthcare sector. Nationwide trajectories have similarly been constructed using prescription data and a similar methodology, where prescription trajectories were constructed from pairs and triads of consecutive prescriptions [22]. Recently, the methodology of [21] was employed on a large cohort of Danish ischemic heart disease patients [23]. The study revealed that the most frequently observed diagnosis codes related to HD patients were manifestations of chronic diseases, with only a few exceptions of more common acute diagnoses such as pneumonia. The combined findings of the considered EHR studies show cardiovascular conditions central to the understanding of multimorbidity. This strongly motivate a further study focusing on chronic conditions among HD diagnosed individuals, which we present in this paper.

Studies such as [21, 23] target the Danish population; however, they are based on registered ICD-10 diagnoses from hospital contacts or outpatient ambulatories. Thus the considered medical diagnoses come from patients with contact to the secondary sector and do not include individuals only receiving care in the primary care sector. Conversely, the constructed prescription trajectories in [22] target the entire Danish population, but chronic diagnoses are not taken into consideration. Therefore, in this study, our analysis will target the population using algorithmic diagnoses [24], utilizing both hospitalization data, prescription data and laboratory service data. Accordingly, we include patients from both the primary and secondary sectors of the Danish health care system. When considering such a high-dimensional and longitudinal data set, patterns related to multiple conditions are complex. Correspondingly and due to their high chronic disease burden, we limit ourselves to a population of HD diagnosed individuals. We consider their disease trajectory, viewing the unique combination of chronic diagnoses, denoted the disease portfolio, as a multimorbidity state. While we limit ourselves to a HD population, our proposed model framework is general and can be applied to study disease portfolio development, using any diagnosis as an index. As such, any multimorbidity patterns revealed are related to the index diagnosis.

Our primary purpose is to map the complex HD trajectories at a population level, presenting diagnosis prevalence and networks of expected disease portfolio development. Secondly, we aim to uncover to what extent factors such as sex, age, social inequality and chronic condition diagnoses influence both speed and direction of the trajectories. As particular combinations of chronic conditions may affect the speed of chronic disease development differently, we study the time between diagnoses, which we denote the diagnosis postponement time to reflect the postponement of new diagnoses, and that the actual onset of chronic disease comes prior to diagnosis. In addition, we study the discrete sequence of diagnoses analyzed in current literature, such as [21, 23].

## Materials and methods

### Data foundation

The data foundation for this analysis is based upon data on sex, age, outpatient clinic visits, hospitalizations, medicine prescriptions, primary sector health services, causes of death, demographics and socioeconomics, which were obtained from the Danish National Patient Registry (NPR) [25], the Danish Psychiatric Central Research Register (PCRR) [26], the Danish National Prescription Registry (DNPR) [27], the Danish National Health Service Registry (NHSR) [28], the Danish register of causes of death [29], the Danish Population Education Register [30] and the Danish Employment Classification Module [31]. The data were linked at an individual level using the unique Danish personal identification number, which is available in the Danish Civil Registration System [32]. As the national registries do not contain data on conditions diagnosed in the primary care sector, we utilized algorithmic diagnoses developed by the Research Center for Prevention and Health at Glostrup University Hospital [24]. This yielded algorithmic diagnosis timestamps for 15 chronic disease diagnoses for the Danish population, covering relevant somatic and psychiatric diseases, which have previously been used in national reports of chronic diagnoses in Denmark [17, 33]. The algorithmic diagnoses process the ICD-10 codes, ATC codes and disease-specific healthcare service utilisations to determine if a particular chronic disease diagnosis was given (S1 Table). Accordingly, the timestamps considered in this study are diagnostic timestamps and should, therefore, not be interpreted as timestamps for disease onsets. Because of this, if ICD-10 codes originating from a hospitalization lasting several days would result in an algorithmic diagnosis, the diagnosis timestamp is taken to be at the midpoint of the hospitalization. Correspondingly, it is possible to obtain multiple chronic diagnoses simultaneously. For each individual, all obtained algorithmic diagnoses at or after the HD diagnosis and until either death, end of the observation period or drop-out due to e.g. emigration are recorded, together with the diagnosis postponement time.

### Study population

The study population contains all Danish citizens aged 18 years or older at some point in time during the period 1/1/1995 to 31/12/2015 who were diagnosed with HD during the observation period (see S1 Table for the specific HD definition).

### A Markov framework

The timescale considered in this study is years following the HD diagnosis. Accordingly, for each individual, the time *t* = 0 is the time at which the HD diagnosis was obtained. Due to the considered diseases chronic nature, we assume that once a diagnosis is obtained, the individual lives with the disease for the rest of their life.

**Fig 1:**
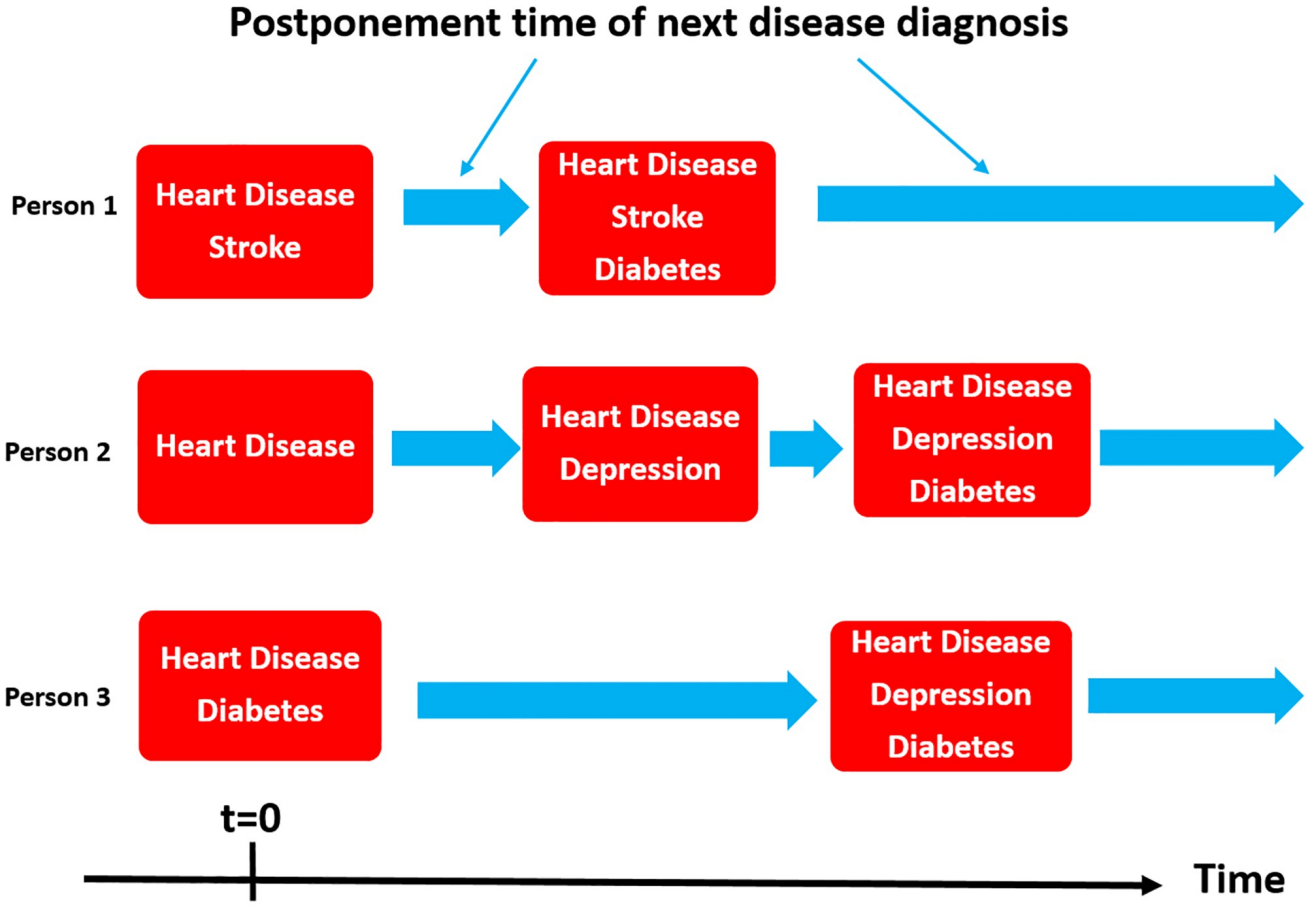
Disease portfolio development. Illustration of disease portfolio trajectories following heart disease diagnosis for three individuals.

Consider the multi-state stochastic process *X*(*t*) describing the development of additional chronic diseases for an individual with HD over time, where *t* ≥ 0 denotes the time in years since the HD diagnosis was decided. The states *s* in the process are disease portfolios of the 15 chronic conditions. Due to the chronic nature of the diseases considered, whenever a change of state occurs in the process, it is always to a state that also includes the chronic conditions of the the previous state. Fig 1 presents an illustration of the process. We can, therefore, always describe the transition probability to a new state through the probabilities of the 15 diseases being the next disease, setting the probability of those in the current disease portfolio to zero. We model {*X*(*t*)}_*t* ≥ 0_ as a continuous-time Markov chain (CTMC), i.e. we assume the Markov property

$$\begin{array}{c} P\left(X\left(t\right)=s_{t}|{\left\{X\left(r\right)\right\}}_{r\leq u}=\left\{s_{r}\right\}\right)=P\left(X\left(t\right)=s_{t}|X\left(u\right)=s_{u}\right),\;u<t \end{array}$$ (1)

Such a CTMC can be characterized by the transition probabilities between states *i* and *j*, *p*_*i*,*j*_ and the exponentially distributed waiting times between state changes; we will focus on the two sub-elements: The discrete-time Markov chain that governs transitions between disease portfolios, and exponentially distributed postponement times, which both through the Markov property only depends on the past through the current portfolio. We thus model postponement times and transition probabilities separately, given a set of individual-level specific explanatory variables, namely the disease portfolio, sex, age, education, occupation and calendar time.

### Statistical analysis

Population characteristics for each sex were calculated at the time when the individual was diagnosed with HD. However, as additional chronic diagnoses are obtained over time, the prevalence of chronic diagnoses is not static. Because of this, we reported the prevalence of each chronic disease by sex at time *t* = 0 and throughout the entire observation period. Additionally, the most prevalent chronic disease portfolios at time *t* = 0 were reported along with a visualization of the diagnosis count distribution of each diagnosis by age.

We right-censored the postponement times when death, drop-out or end of observation period occurred. Initial examination of the postponement time until the following diagnosis verified the exponential distribution of the observations as suitable, thus supporting the Markov assumption. The postponement time for individual *s* when having portfolio ℓ was modelled as exponentially distributed with mean value *μ*_ℓ*s*_ where

$$\begin{array}{c} log\left(\mu_{\ell s}\right)=\sum_{k=1}^{m}\alpha_{k}y_{k\ell s} \end{array}$$ (2)

where *m* (here) determines the total number of explanatory variables, and *y*_*k*ℓ*s*_ is the *k*th explanatory variable originating from individual *s* when having portfolio ℓ. As the explanatory variables contain diagnosis indicators, they can change from portfolio to portfolio for the same individual.

Subsequently, transition probabilities, i.e. the probability *p* of obtaining diagnosis *j* as the following diagnosis provided that individual *s* has portfolio *i* and did not already have the diagnosis *j*, were modelled by logistic regression models:

$$\begin{array}{c} logit\left(p_{ijs}\right)=\sum_{k=1}^{m}\alpha_{k}y_{kijs} \end{array}$$ (3)

with notation similar to (Eq 2). As we were interested in the effects of covariates on the probability of obtaining a specific diagnosis as the next, we worked with separate logistic regressions for each new diagnosis *j*. Specific transition probability estimates can be obtained by normalizing the logit scores.

Model diagnostics were performed and evaluated for the fitted models. For the postponement time model (Eq 2), cox-snell residuals and normalized randomized survival probability (NRSP) residuals [34] were evaluated. For the logistic regression models (Eq 3), deviance residuals were evaluated.

### Variable selection

With our large number of potential effects, we took special care not to over-parameterize the statistical models while maintaining variables with a substantial effect. The selection of explanatory variables considered in the models can be split into two: The selection of dichotomous variables indicating diagnoses and the selection of additional variables representing the biological and social characteristics related to each individual, as well as the calendar time. The explanatory variables, disregarding polynomial and interaction terms, are presented in Table 1. In all analyses, numerical explanatory variables were mean-centered.

**Table 1:** Overview of explanatory variables in the statistical analysis.

Variable(s)	Explanation	Type
Age	The age of the individual in years at time *t* = 0.	Continuous
Calendar time	The calendar time in years at time *t* = 0.	Continuous
Sex	Female/Male, with males as reference level.	Categorical
Education	6 categories (None, Short, Medium, Long, Missing and Missing pre 1920*) with None as reference level.	Categorical
Occupation	8 categories (Retired, Employed, Early retirement pension, Sick leave, Other, Unemployed, Student and Missing with Employed as reference level.	Categorical
Chronic diagnosis	Indicator variables for each of the considered diagnoses.	Dichotomous

*Education information for individuals born previous to 1920 is extremely limited, as most of the information origins from a census in 1970 where individuals older than 50 years were excluded.

To select appropriate interaction effects between the variables in this big-data setting, we performed a comprehensive analysis of both transition probabilities and postponement times, where each distinct disease portfolio was modelled separately. The details are presented in S1 Text. Using the gained knowledge on relevant interaction effects, the postponement time model (Eq 2) and logistic regression models (Eq 3) were estimated, utilizing data from all portfolios. All statistical analyses were performed using the R software version 4.1.3.

### Ethical considerations

The Danish national registries are protected by the Danish Data Protection Act and can only be assessed following application and subsequent approval. No approval from the Danish Research Ethics Committees was needed for this study, since only national registers were used.

## Results and applications

### Descriptive statistics

The data foundation consisted of 6,048,700 Danish citizens aged 18 years or above. In total, 66,986,583 clinical encounters yielded 164,823,811 combined inpatient and outpatient events with assignments of ICD-10 diagnosis codes. Combined with 916,857,561 records of dispensed prescription drugs (ATC medicine codes) and 1,795,102,343 health service records from the primary sector, these diagnosis codes provided the data foundation for chronic disease diagnosis timestamps. The extracted study population included *N* = 766, 596 HD diagnosed individuals. During the observation period, 1,794,403 diagnosis postponement times were observed from 5,021,225 person-years. In total, 9,213 distinct combinations of the 15 diseases were observed, with 1,027,837 transitions between them. The population of HD diagnosed individuals had a mean age of 70.09 ±13.50 years (Table 2). The majority of the population was multimorbid at the time of HD diagnosis, as only 13.71% obtained HD as the first chronic disease. The females in the population were generally diagnosed with heart disease at an older age than males (mean 73.02 vs 67.51 years of age). The multimorbidity level of six or more conditions was more prevalent in females than males. Concerning the prevalence of each of the 15 chronic conditions at time *t* = 0, Table 3 illustrates hypertension, high cholesterol and allergies as common diagnoses obtained at or prior to the HD diagnosis. At time *t* = 0, there was a notable prevalence of hypertension, with males having 64.97% and females having 74.69%. By contrast, the prevalence of high cholesterol was higher among males than females (32.50% vs 23.52%). In addition, it is notable how the prevalence of osteoporosis and long-term use of antidepressants (depression) was higher for females than males (depression 12.30% vs 6.38%; osteoporosis 14.81% vs 4.13%). When considering the lifetime prevalence, the relative increase in the prevalence of psychiatric diagnoses from time *t* = 0 until the end of observation is notable. Dementia, schizophrenia and depression prevalence increased by 319.60, 168.24 and 121.76%, respectively.

**Table 2:** Population characteristics at time of HD diagnosis according to sex.

Variable	Males (n = 406792)	Females (n = 359804)	Total (n = 766596)
Age	67.51 (13.07)	73.02 (13.37)	70.09 (13.50)
Calendar time	2003.55 (6.51)	2003.16 (6.49)	2003.37 (6.50)
Education			
None (≤ 10 years)	30.64 (124658)	36.54 (131480)	33.41 (256138)
Short (11–14 years)	36.77 (149568)	23.26 (83679)	30.43 (233247)
Medium (15–16 years)	7.53 (30619)	4.78 (17191)	6.24 (47810)
Long (≥ 17 years)	5.92 (24094)	2.69 (9682)	4.41 (33776)
Missing	2.25 (9136)	1.66 (5969)	1.97 (15105)
Missing pre 1920	16.89 (68717)	31.07 (111803)	23.55 (180520)
Occupation			
Retired	59.22 (240889)	75.27 (270809)	66.75 (511698)
Employed	26.30 (106992)	11.16 (40144)	19.19 (147136)
Early retirement pension	8.69 (35359)	9.32 (33545)	8.99 (68904)
Missing	0.02 (88)	0.02 (63)	0.02 (151)
Other	1.53 (6236)	1.15 (4120)	1.35 (10356)
Sick leave, etc.	2.55 (10384)	1.91 (6855)	2.25 (17239)
Student	0.17 (711)	0.31 (1102)	0.24 (1813)
Unemployed	1.51 (6133)	0.88 (3166)	1.21 (9299)
Number of chronic disease diagnoses			
1	15.64 (63604)	11.53 (41502)	13.71 (105106)
2	34.79 (141511)	32.45 (116768)	33.69 (258279)
3–5	45.36 (184510)	48.38 (174079)	46.78 (358589)
6+	4.22 (17167)	7.63 (27455)	5.82 (44622)

Values are mean (SD) or % (n).

**Table 3:** Diagnosis prevalence of HD population according to sex. Prevalences are reported at time of HD diagnosis, as well as for the entire span of the observed disease trajectories.

Chronic condition	Male	Female	Total
Prevalence at *t* = 0 (time of HD diagnosis)			
Heart disease	100.00 (406792)	100.00 (359804)	100.00 (766596)
Hypertension	64.97 (264306)	74.69 (268721)	69.53 (533027)
High cholesterol	32.50 (132213)	23.52 (84640)	28.29 (216853)
Allergies	14.51 (59013)	21.16 (76149)	17.63 (135162)
COPD	11.03 (44873)	12.08 (43462)	11.52 (88335)
Diabetes	11.66 (47426)	10.12 (36401)	10.93 (83827)
Depression	6.38 (25955)	12.30 (44255)	9.16 (70210)
Osteoporosis	4.13 (16800)	14.81 (53307)	9.14 (70107)
Stroke	8.81 (35834)	9.06 (32596)	8.93 (68430)
Cancer	6.87 (27934)	7.26 (26122)	7.05 (54056)
Osteoarthritis	5.70 (23193)	8.20 (29496)	6.87 (52689)
Back pain	5.26 (21409)	6.39 (23005)	5.79 (44414)
Dementia	1.39 (5665)	2.58 (9277)	1.95 (14942)
Schizophrenia	1.34 (5466)	1.92 (6926)	1.62 (12392)
Joint disease	1.34 (5455)	1.55 (5580)	1.44 (11035)
Lifetime prevalence^a^			
Heart disease	100.00 (406792)	100.00 (359804)	100.00 (766596)
Hypertension	77.71 (316100)	85.11 (306224)	81.18 (622324)
High cholesterol	51.12 (207983)	37.94 (136502)	44.93 (344485)
Allergies	25.24 (102687)	32.99 (118701)	28.88 (221388)
COPD	25.07 (101975)	24.32 (87506)	24.72 (189481)
Osteoporosis	12.11 (49269)	33.35 (120002)	22.08 (169271)
Stroke	21.15 (86021)	22.12 (79606)	21.60 (165627)
Diabetes	22.74 (92519)	18.75 (67450)	20.87 (159969)
Depression	15.87 (64542)	25.33 (91156)	20.31 (155698)
Cancer	20.38 (82923)	17.92 (64485)	19.23 (147408)
Osteoarthritis	12.63 (51361)	16.47 (59268)	14.43 (110629)
Back pain	11.52 (46846)	13.43 (48332)	12.42 (95178)
Dementia	6.34 (25806)	10.25 (36890)	8.18 (62696)
Schizophrenia	3.56 (14462)	5.22 (18778)	4.34 (33240)
Joint disease	4.26 (17330)	3.73 (13436)	4.01 (30766)

Prevalence are reported as % (n).

^a^Lifetime prevalence is to be understood in the sense that the HD diagnosed individual has been observed to obtain the diagnosis in the time period 1995–2015.

The general high prevalence of hypertension is reflected in the most prevalent disease portfolios at time *t* = 0, as seven of the top ten most frequent portfolios contain hypertension (Table 4). For males and females, the most prevalent portfolio is the portfolio containing a prior hypertension diagnosis (22.51% males, 24.82% females). Interestingly, the fifth most common disease portfolio for females (heart disease + hypertension + osteoporosis) is the 24th most common portfolio for males (prevalence 0.46%), indicating sex differences in the disease portfolio distributions at the onset of HD.

**Table 4:** Top 10 disease portfolio prevalence at time of HD diagnosis according to sex.

	Male		Female		Total	
1	HD + HT	22.51 (91565)	HD + HT	24.82 (89321)	HD + HT	23.60 (180886)
2	HD	15.64 (63604)	HD	11.53 (41502)	HD	13.71 (105106)
3	HD + HT + HC	7.93 (32266)	HD + HT + HC	4.49 (16148)	HD + HT + HC	6.32 (48414)
4	HD + HC	5.86 (23844)	HD + HT + A	3.60 (12938)	HD + HC	3.97 (30461)
5	HD + HT + A	2.30 (9348)	HD + HT + OP	2.21 (7958)	HD + HT + A	2.91 (22286)
6	HD + HT + DI	2.27 (9252)	HD + HT + DI	2.14 (7685)	HD + HT + DI	2.21 (16937)
7	HD + HT + COPD	2.15 (8741)	HD + HC	1.84 (6617)	HD + HT + COPD	2.00 (15357)
8	HD + HT + HC + DI	1.89 (7696)	HD + HT + COPD	1.84 (6616)	HD + HT + HC + DI	1.48 (11372)
9	HD + HT + HC + A	1.54 (6260)	HD + A	1.58 (5676)	HD + A	1.47 (11276)
10	HD + A	1.38 (5600)	HD + HT + HC + A	1.39 (5013)	HD + HT + HC + A	1.47 (11273)

Prevalence are reported as % (n).

HD = Heart Disease, HT = Hypertension, HC = High Cholesterol, A = Allergies, OP = Osteoporosis, DI = Diabetes.

**Fig 2:**
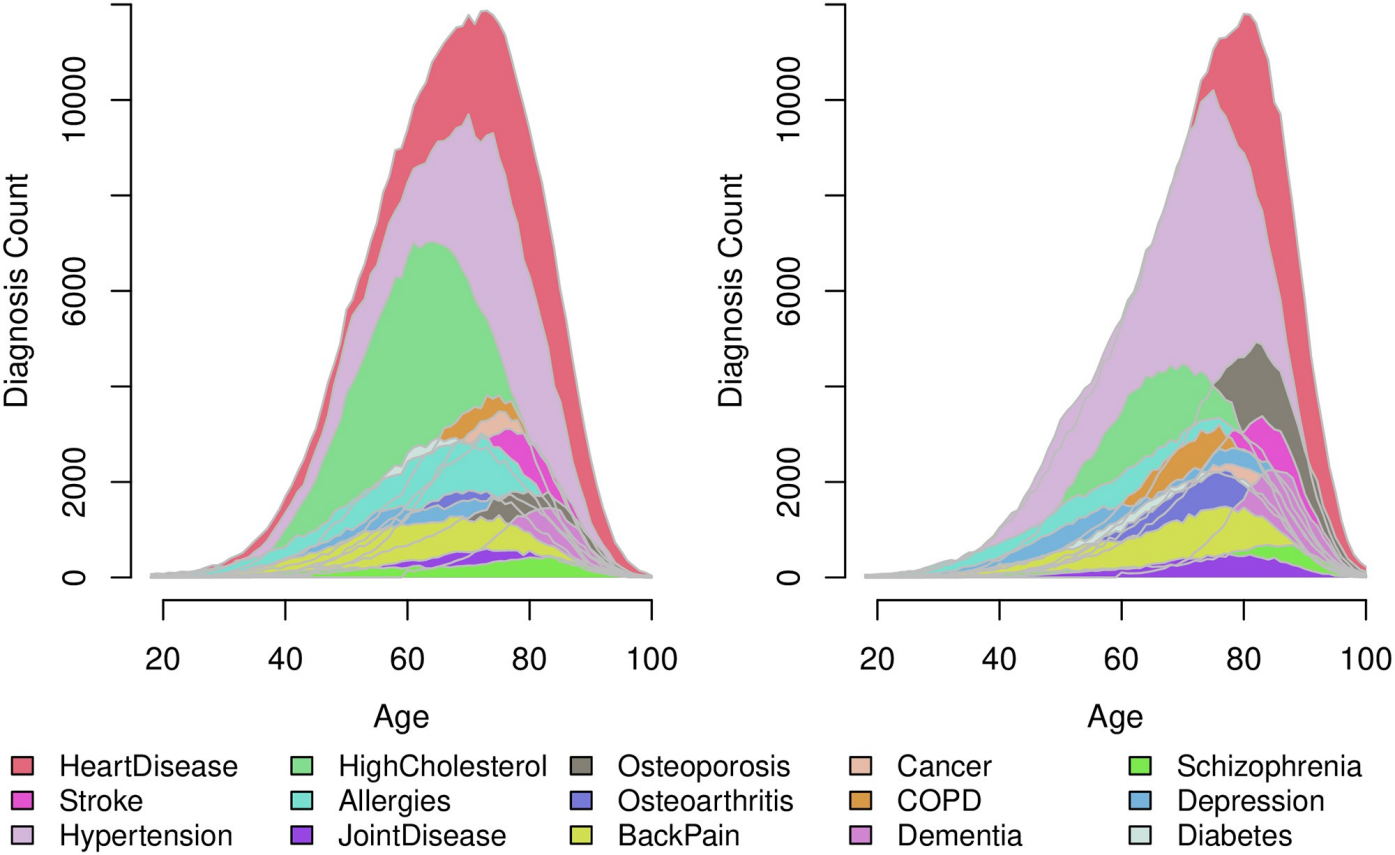
Diagnosis counts by age for the population of Danish chronic heart disease diagnosed individuals in the period 1995–2015. The figure shows males (left) and females (right). A diagnosis is counted when the individual obtains the algorithmic chronic disease diagnosis. The diagnoses are ordered, so diagnoses with larger variance in counts are on top.

Fig 2 shows distributions of the 15 considered chronic disease diagnoses for the chronic heart disease population, stratified by age and sex. The overlap in distributions for a multitude of the considered chronic diagnosis underlines the observed high prevalence of multimorbidity (84.36% males, 88.47% females) at time *t* = 0. In addition, the figure depicts sex-specific differences in diagnosis trends. In particular, a sex difference is observable for the conditions; high cholesterol (green) and osteoporosis (black). The osteoporosis diagnosis predominantly occurs in females. Additionally, the substantially prevalent hypertension diagnosis occurs coincidentally with the HD diagnosis for males. In contrast, for females, both the HD and hypertension distributions are left skewed, with the hypertension distribution skewed marginally to the left of the HD distribution.

### Model-based results

The covariate selection analysis resulted in the removal of several redundant interaction terms. Significant effects in the final postponement time model are shown in Table 5, including significant interactions. Parameter estimates are available in S2 Table. A general socioeconomic gradient is observed with an increasingly positive effect, the higher the achievement of education of the HD diagnosed individual (99% CI Short 0.01–0.04, Medium 0.06–0.11, Long 0.09–0.15). Interestingly, the effects on diagnosis postponement times of hypertension and high cholesterol were positive, although attenuated when both are present in the disease portfolio due to the interaction effect. Any postponement time effect involving cancer was non-significant. The additional diagnoses were significant, but the effect direction largely depended on interactions with additional variables. Back pain and joint disease had an apparent negative effect on the postponement time. Additionally, females had a lower postponement time, compared to males, except when the diagnoses osteoporosis or dementia were prevalent.

**Table 5:** Effects for statistical significant explanatory variables on the postponement time until the next disease diagnosis. Positive and negative effects correspond to increased and decreased diagnosis postponement times.

Variable	Significant effect (P<0.01)	Effect of sublevels, interactions and polynomial effects
Age	Negative	Quadratic effect positive
Calendar time	Mixed	Positive for females, education (short, medium, long), occupation (employed, unemployed), high cholesterol, osteoarthritis, back pain.
		Negative for occupation (early retirement, sick leave, unemployed), hypertension, stroke, diabetes. Quadratic effect negative.
Sex Female	Mixed	Negative effect in general,
		Positive with osteoporosis and dementia.
Education	Mixed	Positive for short (except with diabetes), medium and long.
		Negative for missing (except with diabetes) and missing pre 1920 (except with hypertension or diabetes).
Occupation	Mixed	Positive for employed, other, unemployed.
		Negative for early retirement pension.
Stroke	Mixed	Negative in general. Positive with dementia.
Hypertension	Positive	
High Cholesterol	Positive	
Allergies	Mixed	Positive in general. Negative with high cholesterol.
Joint Disease	Negative	
Osteoporosis	Mixed	Negative in general.
		Positive for females and with back pain, COPD or joint disease.
Osteoarthritis	Mixed	Negative in general.
		Positive for females and with back pain or joint disease.
Back Pain	Negative	
Cancer	No significant effect	
COPD	Mixed	Negative in general. Positive with depression or osteoporosis.
Dementia	Mixed	Negative in general (main effect slightly insignificant).
		Positive for females and with stroke, back pain, or dementia.
Schizophrenia	Mixed	Negative in general. Positive with COPD, schizophrenia or diabetes.
Depression	Mixed	Negative in general. Positive with COPD.
Diabetes	Mixed	Negative in general. Positive with high cholesterol.

**Fig 3:**
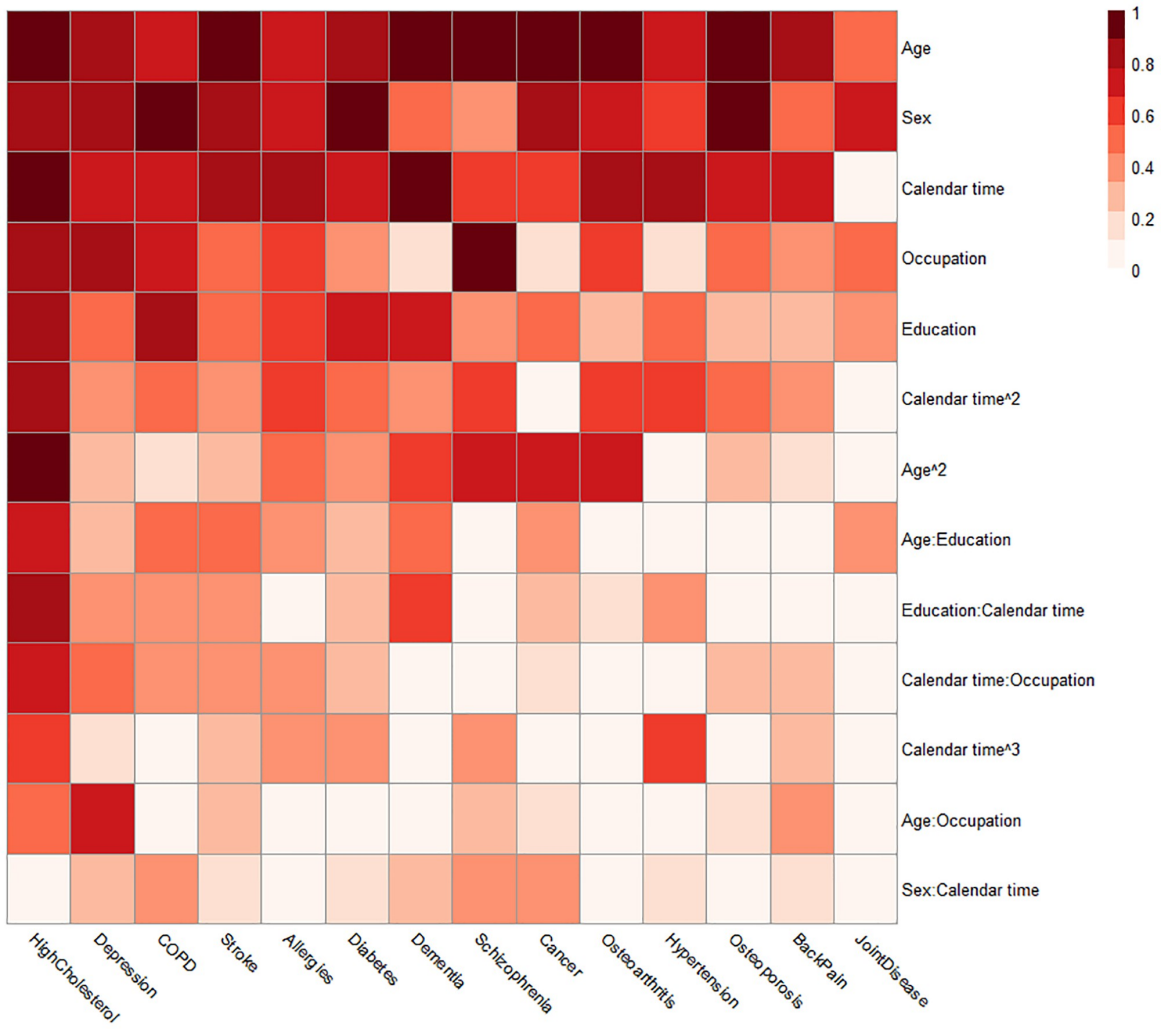
Heatmap on importance of different variables on transition probabilities to distinct next diagnosis endpoints. Results were obtained from a backwards selection of covariates where each unique disease portfolio was modelled separately for each next diagnosis endpoint. The importance of one indicates that the term was kept in all disease portfolios. The figure is sorted by sums of importance across endpoints (left to right) and across variables (top to bottom).

Fig 3 illustrates the result of the backwards selection procedure on the transition probability models across each distinct portfolio and disease endpoints described in S1 Text. A result which stands out is that calendar time is an explanatory variable almost as important as age and sex. Thus, the probability of obtaining a specific diagnosis as the following diagnosis varies across the period considered in the study. Additionally, a substantial amount of variables are needed to account for the probability of obtaining, e.g. high cholesterol and depression, in contrast to diagnoses such as back pain and joint disease. The figure also reveals that the sex of the HD diagnosed individual is particularly important for the risk of osteoporosis, chronic obstructive pulmonary disease (COPD) and diabetes.

By examining the absolute value of the coefficients in the estimated logistic regression models, we rank the importance of each covariate predictor. Table 6 provides an overview of the top five variables with the most considerable discriminative power for each diagnosis endpoint. Sex is a common important variable with considerable discriminative power. For example, the diagnoses stroke, high cholesterol, osteoporosis, cancer, depression and diabetes are heavily influenced by sex, with the female sex having a sizeable positive effect (increased probability relative to alternative diagnoses) on osteoporosis and depression. The common chronic condition hypertension is an important variable for five outcomes and increases the relative probability of stroke, joint disease, COPD, allergies and diabetes. The other common chronic condition, high cholesterol, similarly increases the relative probability of allergies and diabetes along with osteoarthritis and back pain. The explanatory variable important for most chronic condition outcomes is dementia. Dementia is a top predictor for seven distinct diagnoses on its own and has a large positive effect on stroke and schizophrenia as the following diagnosis, with a negative effect for high cholesterol, joint disease, osteoarthritis, back pain, and cancer. Additionally, nine interaction effects involving dementia have great predictive power, including six interactions with stroke (Table 6). Schizophrenia is similarly a condition with great predictive power. It is among the top predictors with a positive effect on the dementia outcome and a negative effect on the joint disease, cancer and high cholesterol outcomes. Additionally, four interactions with schizophrenia are present: two with dementia, one with depression and one with COPD. Finally, osteoporosis positively affects back pain, schizophrenia, and depression, with a negative effect on high cholesterol and diabetes. Exact parameter estimates for the transition probability models are available in S3–S16 Tables.

**Table 6:** Most important variables for transition probabilities. Top five significant (1%) variables with the largest discriminative power across different next diagnosis endpoints as measured by the absolute size of the coefficients in the logistic regression models. The variables are presented by the coefficient sign, where a positive (negative) coefficient corresponds to an increased (decreased) probability of the outcome relative to the other possible outcomes. A colon gives coefficients related to the presence of two simultaneous diseases (Disease 1:Disease 2).

Outcome	Negative effect	Positive effect
Stroke	Female Sex	Missing Education, Dementia, Hypertension, COPD:Schizophrenia interaction
Hypertension		High cholesterol:Diabetes, Dementia:Schizophrenia Back pain:Dementia, Joint disease:Back pain, Stroke:Dementia
High Cholesterol	Dementia, Osteoporosis, Female Sex, Schizophrenia	Missing Education
Allergies		Student Occupation, Stroke:Dementia, Hypertension, Long Education High cholesterol
Joint Disease	Dementia, Schizophrenia	Hypertension, Osteoarthritis, Stroke:Dementia
Osteoporosis		Female Sex, Dementia, COPD, Depression, Cancer
Osteoarthritis	Dementia	High cholesterol, Back pain Joint disease, High cholesterol:Diabetes
Back Pain	Dementia	Osteoarthritis, Osteoporosis High cholesterol, Cancer
Cancer	Dementia, Female Sex, Schizophrenia	Dementia:Schizophrenia, Stroke:Dementia
COPD	Long Education	Stroke:Dementia, Missing Education, High cholesterol:Diabetes, Hypertension
Dementia	Schizophrenia:Depression	Schizophrenia, Missing Education, Depression, Stroke
Schizophrenia	Unemployed Occupation	Dementia, Missing Occupation, Depression, Osteoporosis
Depression		Student Occupation, Stroke, Back pain Female Sex, Osteoporosis
Diabetes	Female Sex, Osteoporosis	Hypertension, High cholesterol, Stroke:Dementia

### Applications

Due to the complexity of the estimated postponement time and transition models, the full effect of specific explanatory variables can be challenging to interpret. We use scenarios to evaluate effects, where we investigate the effect of specific variables by keeping all other variables constant and calculate expected postponement times/transition probabilities. We construct initial disease trajectories for males and females originating from the disease portfolio *HD, hypertension and high cholesterol*, which is a common portfolio at or early after HD diagnosis.

#### A combined effect of degree of multimorbidity on disease postponement time

**Fig 4:**
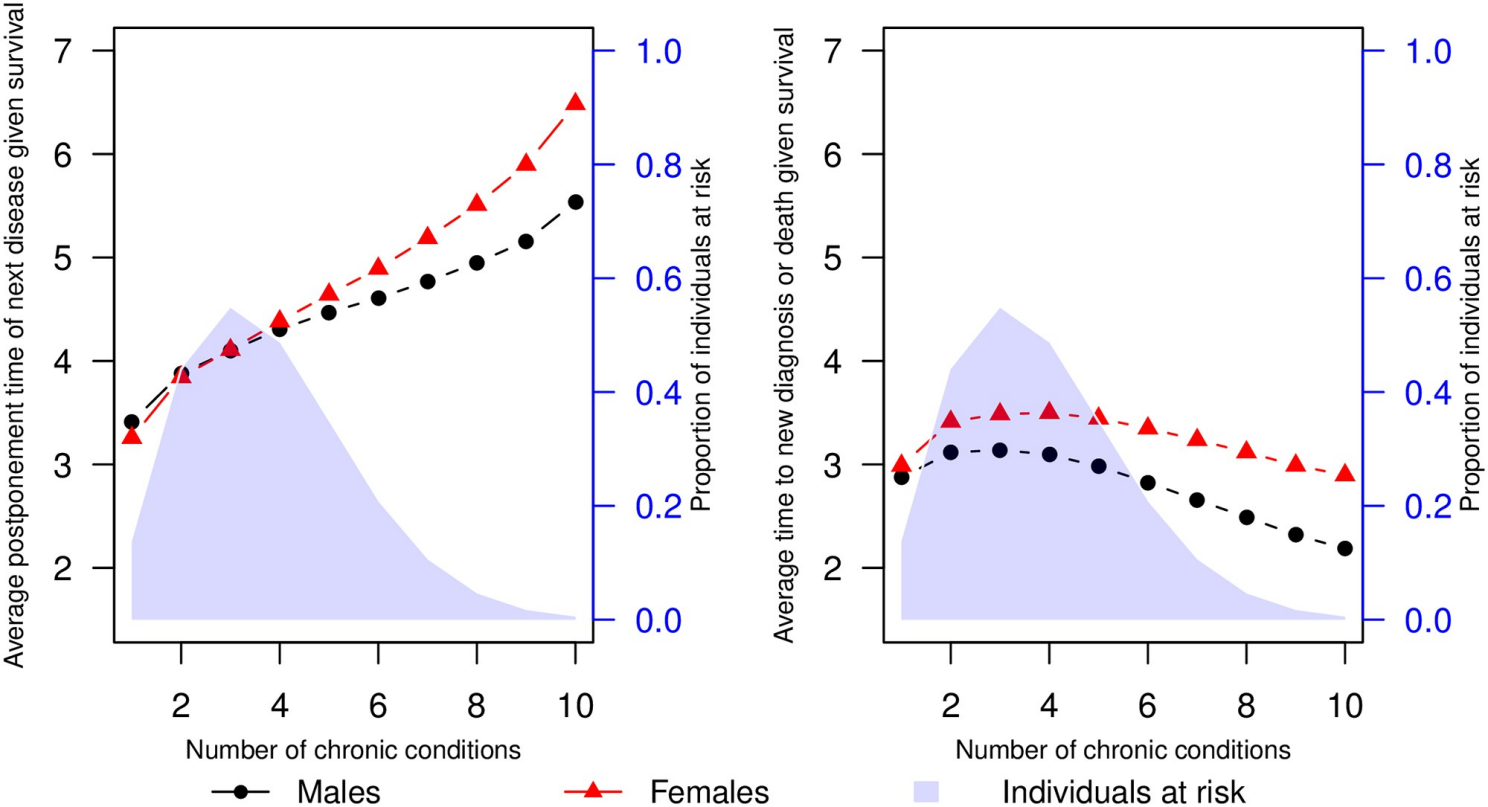
Effect of multimorbidity on postponement times. Estimated mean postponement time of next chronic diagnosis given survival for retired males and females of no education at a set multimorbidity level (left). The mean postponement times are estimated as an average of estimated postponement times for all possible combinations of chronic conditions given the multimorbidity level, weighted by the observed frequency of these combinations. The blue shaded area represents the proportion of HD individuals at risk of a new event. For reference, the right plot represents the same estimates in a model where the new disease event and death are considered a combined event.

We calculated estimated postponement times for all portfolios of size 1–10, separately for retired males and females with no education and with age and calendar time kept constant at their mean levels. The estimated postponement times were subsequently averaged, where each portfolio was weighted by its observed frequency in the HD population. The results are presented in Fig 4 along with comparative results from a separate model, where disease postponement time was replaced by the postponement time to either a new diagnosis event or death. A shaded blue region represents the proportion of individuals in the study observed to have a specific level of multimorbidity. In general, an increased postponement time is observed for increasing levels of multimorbidity. However, the proportion of individuals at risk of a new diagnosis is dramatically reduced for multimorbidity levels above four. Postponement times are marginally greater for males than females, except for individuals with more than six diagnoses. Considering the postponement time to new diagnosis or death, an initial increase is observed with multimorbidity levels lower than 5 diagnoses, from which the postponement time decreases for greater levels of multimorbidity (5+ diagnoses). The postponement time to new diagnosis or death is generally greater for males than females, with the difference between sexes most distinct at high multimorbidity levels (6+ diagnoses).

#### Disease trajectories by sex and education

**Fig 5:**
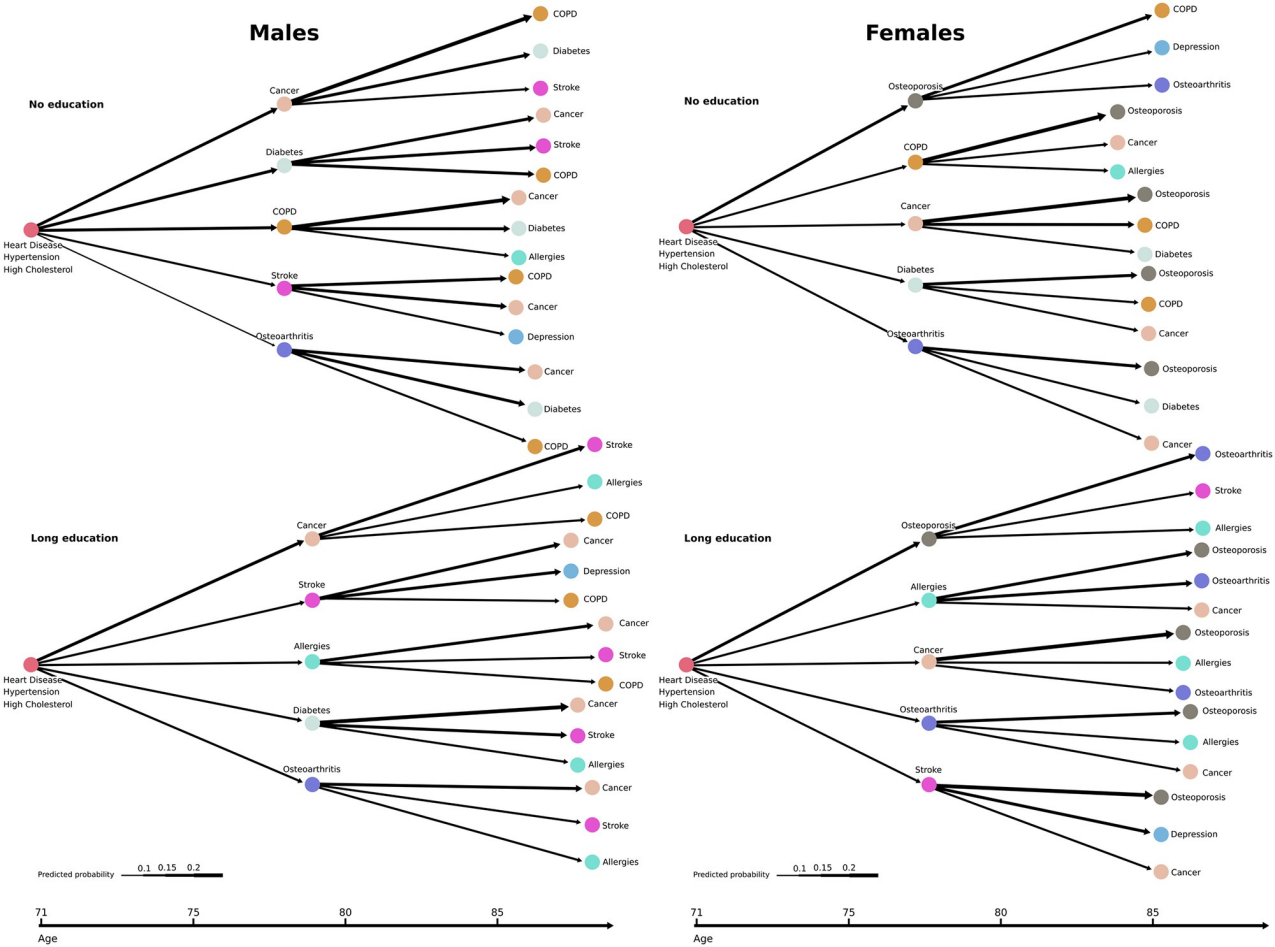
Sex-specific disease trajectories contrasting educational attainment. Disease trajectories starting from the most common triad of diseases for males (left) and females (right) of no education (top) and long education (bottom). Trajectories are constructed for retired individuals with calendar time and age set at the mean levels at *t* = 0 (70.09 years of age and 2003.37 year time, respectively). The length of the arrows corresponds to the modelled diagnosis postponement time. The width of the arrows corresponds to the modelled probability of obtaining the diagnosis next.

Starting from the portfolio HD, hypertension and high cholesterol at time *t* = 0, we constructed disease trajectories from the Markov framework and the estimated parameters in Eqs (2 and 3), separately for males and females at different educational attainment levels. The occupation level was kept at retired, with calendar time and age set at the mean levels at *t* = 0 (70.09 years of age and 2003.37 year time, respectively). The five diagnoses with the highest assigned transition probability in the first stage and the three diagnoses with the highest probabilities in the second stage for each of the first stage diagnoses are presented in Fig 5 for combinations of males/females and no/long education. Cancer, diabetes and COPD appear as common diagnoses in the first and second stages (i.e., first and second diagnoses obtained from the initial portfolio) for males and females with no education. These diagnoses generally appear around the same age as the heart disease diagnosis (males/females mean age of cancer 73.03/74.18, COPD 70.65/72.03, diabetes 65.86/70.48, HD 67.50/73.02 years of age, Fig 2). Apart from these, the diagnosis trajectories differ considerably by sex when considering the types of next diagnoses (Fig 5). The male trajectory encompasses a high appearance of cancer, with the female trajectory a high appearance of osteoporosis. Interestingly, the psychiatric diagnosis of depression appears in both the male and female trajectories following a stroke diagnosis (except for females of no education). For females of no education, however, stroke is not among the five most likely stage 1 diagnoses, and the diagnosis of depression appears here following osteoporosis.

**Fig 6:**
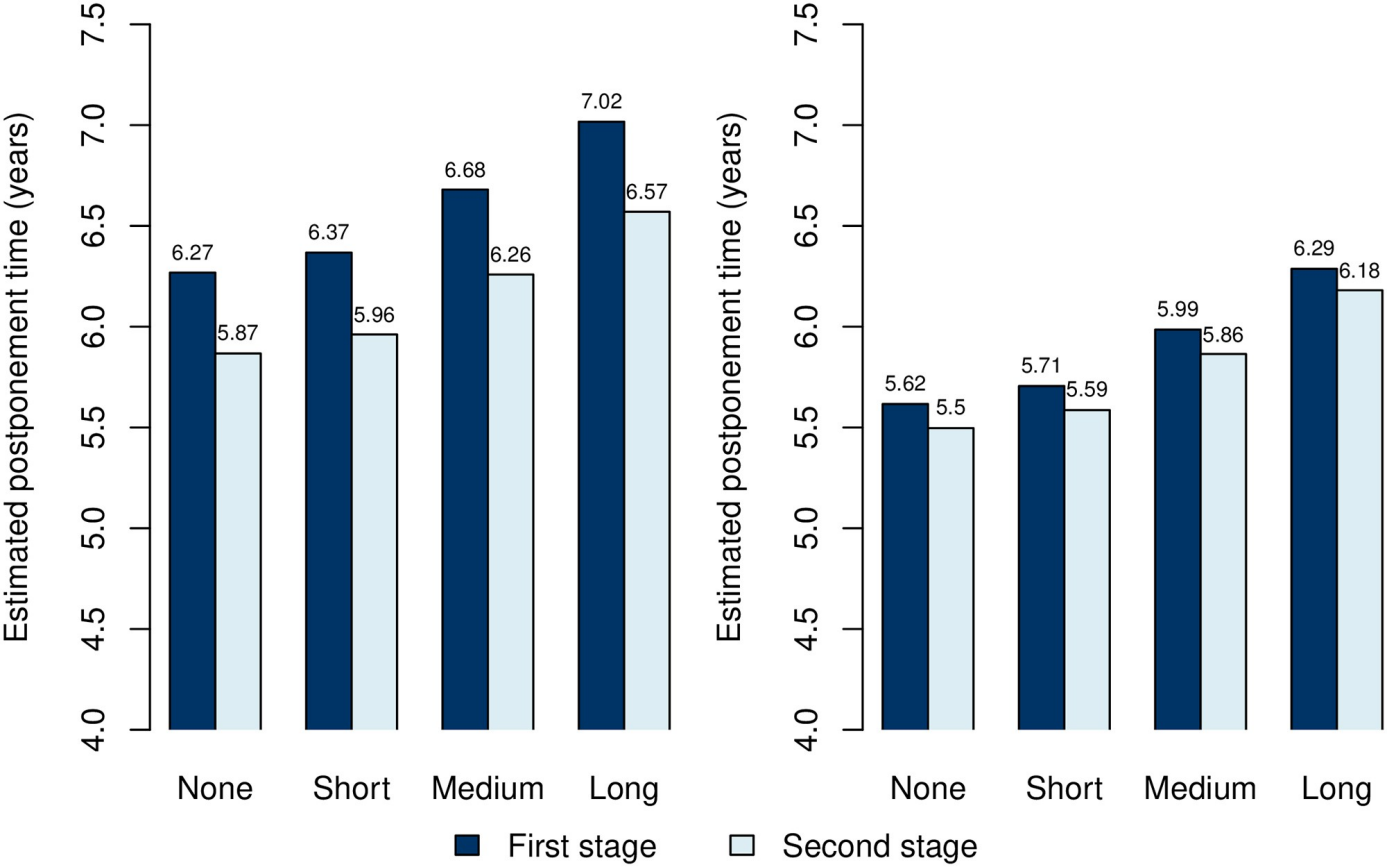
Estimated mean postponement times by educational attainment levels. The figure presents the mean postponement time of the next chronic diagnosis given survival for retired males (left) and females (right) at various educational levels. Estimations are made starting from the most common triad of diagnoses heart disease, hypertension, and high cholesterol (see Fig 5). The second stage postponement times are a weighted average with weights determined by the transition probabilities of the first stage diagnoses.

Regarding the illustrated postponement times, the time between diagnoses is generally longer for individuals with long education compared to those without education, especially in the second stages. Additionally, obtaining either COPD or stroke in the first stage substantially decreases the postponement time in the second stage for both males and females. Fig 6 illustrates the first and second stage postponement times at four increasing educational attainment levels. A general increase in postponement times with educational attainment is observed. Moreover, following the most common triad of diagnoses, the postponement time for males decreases from the first to the second stage. This observation is less distinguishable for females. Additionally, the postponement time is generally shorter for females compared to males. Trajectories segregated by sex for the four considered educational attainment levels are available in S1–S4 Figs.

## Discussion

When ageing, multimorbidity is becoming more the rule rather than the exception [3, 18, 21, 35–37]. As a result, the management of individuals with numerous chronic conditions is a global challenge for society and healthcare systems. There is a need for better understanding of the epidemiology of multimorbidity in order to obtain the most successful healthcare and societal trajectories and interventions. This is central for heart disease diagnosed individuals, as cardiovascular disease clusters are among the most common forms of multimorbidity [21, 37]. In this study, we use a data-driven Markov model approach to characterize longitudinal multimorbidity patterns through the progression of disease portfolios. This is done in a large population derived from register records collected over two decades covering the entire Danish population using heart disease as an index disease. Gaining insight into the structure of disease portfolios is in itself a relevant outcome, and uncovering portfolios with specific combinations of diagnoses which play a significant role in the development of the trajectories is a step towards understanding the epidemiology of multimorbidity within HD populations and may provide insights for secondary analyses and hypotheses regarding underlying pathologies between diseases.

### Main findings

This large-scale study aimed to map the multimorbid chronic disease trajectories for individuals with chronic heart disease and explore associations between these trajectories and explanatory factors for further development of diagnoses at a general population level. We have revealed specific chronic condition patterns to significantly influence the risk for the development of additional diagnoses, both according to the type of condition and time until diagnosis. Our mapping of the HD trajectories revealed that they are highly multimorbid, as shown by the lifetime prevalence of all but three diagnoses (dementia, schizophrenia, joint disease) exceeding 10% (Table 3). We have illustrated typical sex-segregated trajectories, where female trajectories were dominated by osteoporosis and male trajectories by cancer, which along with diabetes, were also prevalent for females. We found sex to be important for the development of almost all chronic conditions (Fig 3), in particular stroke, high cholesterol, osteoporosis, cancer, depression, COPD and diabetes (Table 6, Fig 3). Additionally, we found the female sex to be associated with shorter diagnosis postponement times (Table 5), indicating an increased speed of pathophysiology giving rise to faster new diseases or faster diagnosis caused by increased awareness in the healthcare system and by the single patient. Our mapping further showed clear social disparities in the multimorbid HD trajectories. A socioeconomic gradient was observed where diagnosis postponement time increases with educational attainment (Fig 6). Additionally, contrasts in diagnosis development based on educational levels were observed for both sexes. The contrasts were manifested by increased probabilities relative to other diagnoses for severe diagnoses such as COPD and diabetes at the lowest educational attainment level compared to the highest.

### Disease portfolios and chronic diagnoses

The importance of taking the complete chronic disease portfolio of the HD diagnosed individual into consideration when evaluating the development of diagnoses is supported by the significant effects of the chronic diagnoses on both postponement times and transition probabilities and, in particular, their mutual interactions and interactions with explanatory variables such as age and gender (Tables 5 and 6, S2–S16 Tables). These strong interaction effects indicate that the HD diagnosed individuals’ trajectories are heavily complicated by multimorbidity. Due to the effects of the interactions, the individual should not be evaluated on a single diagnosis basis as the diagnosis postponement time, and the probability of obtaining any diagnosis as the next depends on the combination of diagnoses already present. Instead, the individual’s complete disease portfolio should be carefully evaluated when considering treatments and possible prevention health services, in order to avoid single diagnosis silo-based thinking.

We found dementia to be important for the majority of diagnosis outcomes (Table 6), despite the relative low prevalence of dementia within the HD population (Table 3). This finding may suggest that dementia is a highly complex condition in HD diagnosed individuals, greatly affecting the trajectories whenever present.

Depression was found to commonly occur following stroke in the illustrated disease trajectories (Fig 5, S1–S4 Figs). Post-stroke depression is common and associated with adverse outcomes such as higher mortality, worse recovery and lower quality of life [38]. Our results show that post-stroke depression manifests itself in the chronic heart disease trajectories.

It is crucial to discuss how the effects of the transition probability models should be interpreted. Taking an example from Table 6; when the female sex has a negative effect on the stroke outcome, it means that there is a lower risk of obtaining stroke as the next diagnosis, relative to any other diagnosis for females, compared to males. This does not mean that the risk of stroke is low for females; it is simply greater for men (Fig 5). Similarly, for positive effects, the risk of obtaining allergies as the next diagnosis, relative to alternative diagnoses, is increased if hypertension is present in the portfolio. This does not necessarily suggest a direct association between the two diagnoses. It may be because in some cases where hypertension is present, allergy is more frequently the next diagnosis due to a healthier HD diagnosed individual, who is not at immediate risk of some of the more severe diagnoses. Taking the diagnosis cancer as an example, the trajectory networks illustrated a greater estimated risk, compared to alternative diagnoses (Fig 5, S1–S4 Figs). Cancer generally occurring in the older population (Fig 2) may explain this relative importance, as opposed to a direct association between HD and cancer.

In Fig 4, the frequency weighted mean postponement time until a new diagnosis was illustrated to increase with multimorbidity level, with all other explanatory factors kept constant. It is crucial to consider the population of HD diagnosed individuals at risk. The number of individuals at risk at greater multimorbidity levels (5+ or more diagnoses) is markedly declining. Additionally, there is a decreasing trend in event times for this selected population when considering the combined endpoint of new diagnosis or death. The few people who get a new diagnosis are subject to a survivorship bias, which may explain that their postponement times until a new diagnosis are longer.

Interestingly, the disease postponement time effect of both hypertension and high cholesterol were positive (Table 5), indicating an increased time until the next diagnosis compared to not having the diagnosis. The two diagnoses are commonly known risk factors for heart disease [39–41] and, consequently, occur frequently in HD diagnosed individuals. From Table 3, it is observable that a large proportion of the HD population already have either of the diagnoses at the time of HD diagnosis, with a large proportion of the population obtaining the diagnoses throughout their lifetime. Therefore, if the HD diagnosed individual does not already have either the hypertension or high cholesterol diagnosis, the subject is likely to obtain either as the following disease (S3–S16 Tables). Accordingly, the postponement time is shorter when these diagnoses are not present, as there is a fast movement towards primary risk factors if they are not already present.

On the notion of diagnoses, it is not the authors’ viewpoint that all of the included chronic diagnoses are natural, biological comorbidities linked to the heart disease diagnosis (see [42] for a discussion of comorbidity vs multimorbidity). Some diagnoses commonly occur around the same time in life as heart disease. Nevertheless, because HD is the index diagnosis in our investigation, some of the multimorbidity patterns that were found are to some extent dependent on the HD diagnosis. Following Boyd’s definition of multimorbidity as ‘the coexistence of two or more chronic conditions, where one is not necessarily more central than the others’ [43], we study longitudinal multimorbidity patterns in relation to HD diagnosed individuals.

### Sex related differences

The study found apparent sex-related differences in disease trajectories for both transitions to new diagnoses and postponement times. In general, the postponement time sex effect resulted in faster development of new diagnoses for females compared to males. However, for high multimorbidity levels (7 or more diagnoses), the reverse case was observed (Fig 4). For transition probabilities, sex was important for the majority of next diagnosis endpoints (Fig 3). The osteoporosis diagnosis notably appeared to dominate the female HD trajectories, as opposed to the male trajectories, where the relative probability for cancer and COPD as the following disease was generally higher. In the ischaemic heart disease study [23], the osteoporosis diagnosis was primarily found as a diagnosis obtained late in ischaemic heart disease trajectories and was not only attributed to the female sex. Nevertheless, osteoporosis is commonly known to become increasingly prevalent in ageing females [44], and our study revealed that it predominated female but not male trajectories following HD diagnosis. Compared to females, male trajectories contained stroke to a greater extent across different socioeconomic levels (Fig 5). Previous studies have shown a reduced risk of stroke following heart failure for females compared to males [45], despite the general association between stroke and heart disease [13, 46, 47]. In our study, this is reflected in the stroke condition appearing more often in the disease trajectories for males compared to females.

### Social disparities

Investigating the impact of social inequality on HD portfolio trajectories may uncover general social disparities and contrasts in disease development. Understanding the manifestation of social inequality within multimorbidity and its relation to specific portfolios is therefore crucial in the derivation of new knowledge. Our study found social disparities in the HD disease trajectories, particularly regarding the diagnosis postponement times, with a clear social gradient (Table 5, Fig 6, S2 Table). For both sexes, the longer the education, the longer the HD diagnosed individual postpones the following diagnosis. Prevalence of multimorbidity has previously been documented to be inversely related to educational attainment in cross-sectional studies [10, 48], which for HD diagnosed individuals possibly can be attributed to the slowing of diagnosis development, the higher education attainment. Social disparities were also found in the transitions to new diagnoses (Fig 5). In general, as the educational level increases, COPD becomes less common as the next disease for both females and males. Conversely, stroke and allergies are diagnoses that, relative to the other diagnoses, become more common with higher education levels. Allergies, a less severe diagnosis compared to some of the alternatives, being more prevalent compared to other illnesses at higher educational levels, may indicate a healthier state of the well-educated HD diagnosed individuals. The risk of diabetes as the next diagnosis, relative to alternatives, similarly decreases with educational attainment. In general, lifestyle-related diagnoses such as diabetes (we consider both type 1 and type 2) and COPD occur with a lower probability in the trajectories as the educational level increases. This may be attributable to a less healthy lifestyle in the lower socioeconomic groups [49]. In particular, risk factors for COPD, such as smoking, has also been linked to the development of type 2 diabetes [50] and can possibly explain why those two diagnoses appear in the trajectory of the lowest educational attainment level.

### Calendar time

When studying multimorbidity patterns over time, we argue that calendar time is essential to be adjusted for. This is reflected in the relative importance of calendar time when determining possible next chronic disease diagnoses (Fig 3, S3–S16 Tables). These findings could indicate that medical diagnostic procedures have changed between 1995 and 2015 and/or that chronic disease treatment and prevention in society has improved. This phenomenon is possibly attributable to the development of more accurate and cheaper diagnostic methods for specific diagnoses and/or progression in societal prevention. A large systematic review and meta-regression analyzing mortality and readmissions among acute heart failure diagnosed patients [51] concluded favourable survival trends after heart failure in the period 1980 through 2017, suggesting improved disease management for some parts of the heart diseases considered in our study.

### Strengths and limitations

The main strength of our study is the large population, consisting of all heart disease diagnosed adults in the Danish population from 1995–2015. The study population is thus not a sample, which obviates any concerns for representability. Generally, data from the Danish national registers are of high quality, with reliable, complete information [52]. To the best of our knowledge, this study is the most extensive longitudinal study of heart disease-diagnosed individuals, including comprehensive information about both somatic and psychiatric diagnoses, educational attainment and employment status. While the ischaemic heart disease population considered in [23] is largely overlapping the population in our work, we include socioeconomic information, algorithmic diagnoses, and a broader range of heart disease diagnoses, recognizing the heterogenicity of pathophysiology lying behind the different categories of heart diseases [41, 53]. In addition, including diagnosis information from the primary care sector through algorithmic diagnoses allows for conclusions based on a general population instead of looking at individuals who have been in contact with a hospital. Despite this, studies working solely with hospital encounters are informative [21, 23], as specific conditions can be examined at the precise ICD-10 diagnosis level, in contrast to the broader, more general definitions of conditions considered in this study.

There are several limitations associated with this study. Although the algorithmic diagnoses considered in this study previously have been shown to be reliable [24], using them in a longitudinal setting introduces the question of the exact time when the medical diagnosis was decided. Additionally, the criteria for obtaining a particular diagnosis can change over time, which further complicates timestamp extraction. The proper timestamp of chronic disease onset comes prior to diagnosis, particularly a hospital encounter or medicinal-based diagnosis. Consequently, we have stressed using the term ‘diagnosis postponement time’ instead of terms such as ‘waiting times’ between diseases. Regarding postponement times, it is essential to mention that while a shorter postponement time corresponds to an increased speed of the subsequent diagnosis, it does not strictly imply faster new manifestations of diseases. The short postponement time might be due to awareness, either in the healthcare system or personally. Regarding sex-related differences, females having shorter postponement times might be due to such personal awareness.

In this study, a large group of individuals had missing educational attainment information. We chose to model the missing values as separate categories, as the data were not missing at random. We experimented with random-forest-based imputations [54], which led to similar results as those presented. A challenge when considering chronic diseases over a time horizon is the very definition of what is meant by the word ‘chronic’. Our approach of considering individuals living with all of the considered diagnoses for the rest of their lives, once it is obtained, can be challenged for some diagnoses (e.g. some types of cancer).

### Future work

Future work could allow transitions to states removing conditions based on not having observed a related prescription or ICD-10 diagnosis for a particular period. This, however, would heavily bias the postponement times between diagnoses. This work concerns prospective analysis from the HD diagnosis. However, one could also investigate the behavior of the portfolio process backwards in time from *t* = 0, i.e., which portfolios lead up to the HD diagnosis and when. This analysis is technically more complicated in nature because we will need to condition on that all diagnoses are made between the age at HD diagnosis and the age 0, i.e. birth, meaning that one must model a bridge between two portfolios, of which the one at *t* = 0 may vary. We have deferred this analysis to further research.

We want to stress the generality of our proposed methodology. In this work, we have examined heart disease trajectories. However, it is possible to condition on any particular chronic disease as an index diagnosis to study multimorbid trajectories related to the index. This enables similar studies of longitudinal multimorbidity trends within selected chronic disease populations. The methodology proposed in this work included exponential postponement times while modelling transitions by logistic regressions. These models allowed for interpretations of their coefficients, which enabled us to outline factors influencing the development of the disease trajectories. Much work has been put into predictive models, primarily deep-learning-based models using either a long short term memory [55], or transformer design [56]. Such models generally achieve great predictive power but are more challenging to interpret due to their large number of parameters and do not provide effect parameters.

## Conclusion

Our results emphasize that the disease trajectories of individuals diagnosed with chronic heart disease are heavily complicated by multimorbidity. We argue it is crucial to consider and study chronic heart disease patients’ entire disease portfolio. Furthermore, our longitudinal analysis of the diagnosis sequences and the intervals between them identified several factors crucial to the trajectories’ speed and direction. We identified sex-based differences in the trajectories. Compared to males, female trajectories received new diagnoses faster, and osteoporosis and psychiatric diagnoses caused complications for them to a greater extent. Furthermore, social disparities were similarly observed, manifested in a clear social gradient where the higher educational attainment the HD diagnosed individual has, the slower the development of new chronic diagnoses. Additionally, we highlight a socioeconomic contrast in diagnosis development. Compared to people with high educational levels, people with low educational attainment have trajectories that are more heavily influenced by lifestyle-related diagnoses such as COPD and diabetes. Finally, in a world where multimorbidity is on the rise, our findings demonstrate the importance of national data-driven analyses of disease portfolios. Our proposed Markov framework can be applied conditioning on any index diagnosis, studying the progression of multimorbidity. This is a valuable tool in order to understand the epidemiology of multimorbidity, creating a healthcare system organized to handle individuals with multiple chronic diseases.

## Supporting information

S1 Fig Disease trajectories at the no educational attainment level. Disease trajectories starting from the most common triad of diseases for males (top) and females (bottom). Trajectories are constructed for retired individuals with no education, and calendar time and age set at the mean levels at *t* = 0 (70.09 years of age and 2003.37 year time, respectively). The length of the arrows corresponds to the modelled diagnosis postponement time. The width of the arrows corresponds to the modelled probability of obtaining the diagnosis next.
(TIF)

S2 Fig Disease trajectories at the low educational attainment level. Disease trajectories starting from the most common triad of diseases for males (top) and females (bottom). Trajectories are constructed for retired individuals with no education, and calendar time and age set at the mean levels at *t* = 0 (70.09 years of age and 2003.37 year time, respectively). The length of the arrows corresponds to the modelled diagnosis postponement time. The width of the arrows corresponds to the modelled probability of obtaining the diagnosis next.
(TIF)

S3 Fig Disease trajectories at the medium educational attainment level. Disease trajectories starting from the most common triad of diseases for males (top) and females (bottom). Trajectories are constructed for retired individuals with no education, and calendar time and age set at the mean levels at *t* = 0 (70.09 years of age and 2003.37 year time, respectively). The length of the arrows corresponds to the modelled diagnosis postponement time. The width of the arrows corresponds to the modelled probability of obtaining the diagnosis next.
(TIF)

S4 Fig Disease trajectories at the high educational attainment level. Disease trajectories starting from the most common triad of diseases for males (top) and females (bottom). Trajectories are constructed for retired individuals with no education, and calendar time and age set at the mean levels at *t* = 0 (70.09 years of age and 2003.37 year time, respectively). The length of the arrows corresponds to the modelled diagnosis postponement time. The width of the arrows corresponds to the modelled probability of obtaining the diagnosis next.
(TIF)

S5 Fig Overview of covariate interaction selection. (TIF)

S1 Table Algorithmic diagnoses. Algorithms used to define the 15 conditions.
(DOCX)

S2 Table Parameter estimates for effects on the postponement time until a new chronic disease diagnosis. (DOCX)

S3 Table Parameter estimates for effects on obtaining stroke as the next chronic disease diagnosis. (DOCX)

S4 Table Parameter estimates for effects on obtaining hypertension as the next chronic disease diagnosis. (DOCX)

S5 Table Parameter estimates for effects on obtaining high cholesterol as the next chronic disease diagnosis. (DOCX)

S6 Table Parameter estimates for effects on obtaining allergies as the next chronic disease diagnosis. (DOCX)

S7 Table Parameter estimates for effects on obtaining joint disease as the next chronic disease diagnosis. (DOCX)

S8 Table Parameter estimates for effects on obtaining osteoporosis as the next chronic disease diagnosis. (DOCX)

S9 Table Parameter estimates for effects on obtaining osteoarthritis as the next chronic disease diagnosis. (DOCX)

S10 Table Parameter estimates for effects on obtaining back pain as the next chronic disease diagnosis. (DOCX)

S11 Table Parameter estimates for effects on obtaining cancer as the next chronic disease diagnosis. (DOCX)

S12 Table Parameter estimates for effects on obtaining COPD as the next chronic disease diagnosis. (DOCX)

S13 Table Parameter estimates for effects on obtaining schizophrenia as the next chronic disease diagnosis. (DOCX)

S14 Table Parameter estimates for effects on obtaining dementia as the next chronic disease diagnosis. (DOCX)

S15 Table Parameter estimates for effects on obtaining depression as the next chronic disease diagnosis. (DOCX)

S16 Table Parameter estimates for effects on obtaining diabetes as the next chronic disease diagnosis. (DOCX)

S1 Text Supplementary methods. (PDF)
