# Correction: Longitudinal models for the progression of disease portfolios in a nationwide chronic heart disease population

**DOI:** 10.1371/journal.pone.0304245

**Published:** 2024-05-17

**Authors:** Nikolaj Normann Holm, Anne Frølich, Ove Andersen, Helle Gybel Juul-Larsen, Anders Stockmarr

In the Author Contributions, Nikolaj Normann Holm should be listed as one of the persons who wrote the paper.
