# Correction: Longitudinal models for the progression of disease portfolios in a nationwide chronic heart disease population

**DOI:** 10.1371/journal.pone.0308820

**Published:** 2024-08-08

**Authors:** Nikolaj Normann Holm, Anne Frølich, Ove Andersen, Helle Gybel Juul-Larsen, Anders Stockmarr

The plots are incorrect in Fig 4. Please see the correct Fig 4 here.

**Fig 4:**
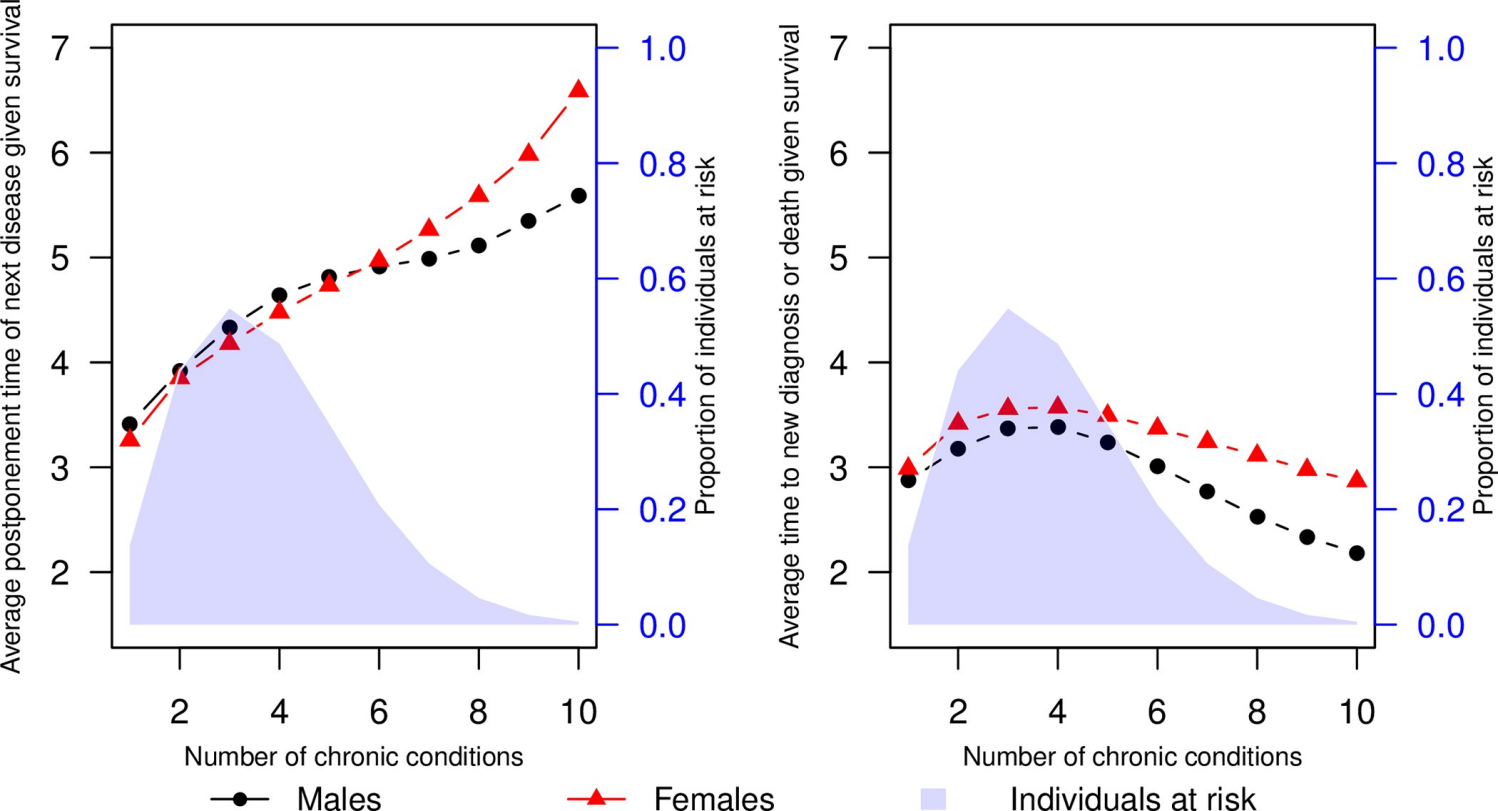
Effect of multimorbidity on postponement times. Estimated mean postponement time of next chronic diagnosis given survival for retired males and females of no education at a set multimorbidity level (left). The mean postponement times are estimated as an average of estimated postponement times for all possible combinations of chronic conditions given the multimorbidity level, weighted by the observed frequency of these combinations. The blue shaded area represents the proportion of HD individuals at risk of a new event. For reference, the right plot represents the same estimates in a model where the new disease event and death are considered a combined event.

In the subsection titled “A combined effect of degree of multimorbidity on disease postponement time,” which is within the section titled “Results and Application,” there is an error in the ninth sentence. The correct sentence is: The postponement time to new diagnosis or death is generally lower for males than females, with the difference between sexes most distinct at high multimorbidity levels (6+ diagnoses).

In S1 Table, there is an error in the row labeled “Heart disease” and in the row labeled “Dementia.” In the row labeled “Heart Disease,” the values in the column labeled “ICD-10 from NPR and PCRR” should have been “I20, I21, I23-I25, I50, I11, I13.” In the “Dementia” row, the text in the column labeled “Definition” should have been “(DIAG)a All patients aged 60 years or older at contact. And/or (MEDICINE)b with ATC: N06D, also aged 60 years or older at the date of distribution.” Please see the correct S1 Table here.

## Supporting information

S1 Table Algorithmic diagnoses. Algorithms used to define the 15 conditions. (DOCX)
