# Supplementary figures and images for: Longitudinal models for the progression of disease portfolios in a nationwide chronic heart disease population
Source: PLoS One. 2023 Apr 20;18(4):e0284496. doi: 10.1371/journal.pone.0284496 (PMC10118194; doi:10.1371/journal.pone.0284496)

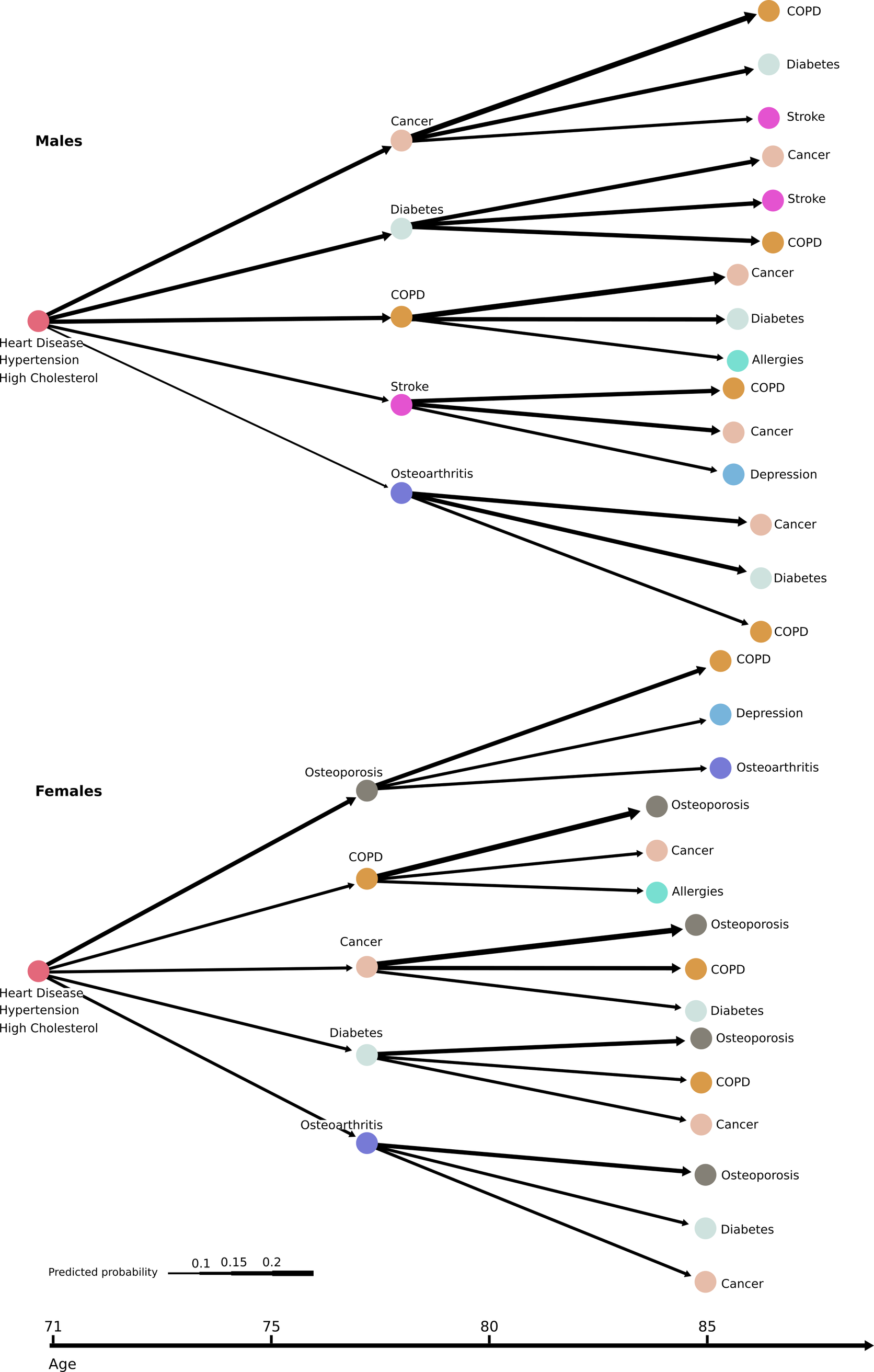

Supplement: S1 Fig — Disease trajectories starting from the most common triad of diseases for males (top) and females (bottom). Trajectories are constructed for retired individuals with no education, and calendar time and age set at the mean levels at t = 0 (70.09 years of age and 2003.37 year time, respectively). The length of the arrows corresponds to the modelled diagnosis postponement time. The width of the arrows corresponds to the modelled probability of obtaining the diagnosis next. (TIF) [file pone.0284496.s001.tif]

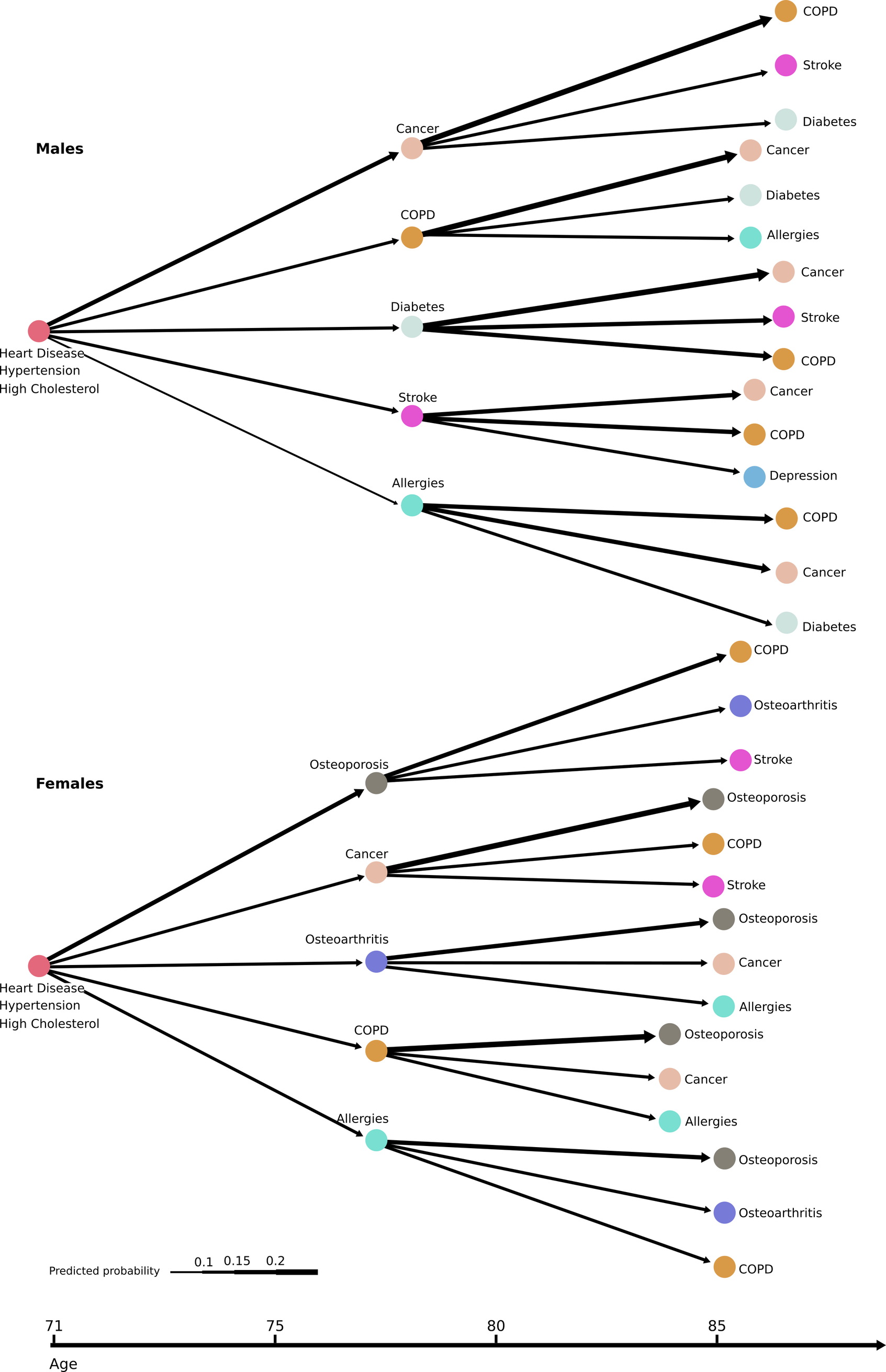

Supplement: S2 Fig — Disease trajectories starting from the most common triad of diseases for males (top) and females (bottom). Trajectories are constructed for retired individuals with no education, and calendar time and age set at the mean levels at t = 0 (70.09 years of age and 2003.37 year time, respectively). The length of the arrows corresponds to the modelled diagnosis postponement time. The width of the arrows corresponds to the modelled probability of obtaining the diagnosis next. (TIF) [file pone.0284496.s002.tif]

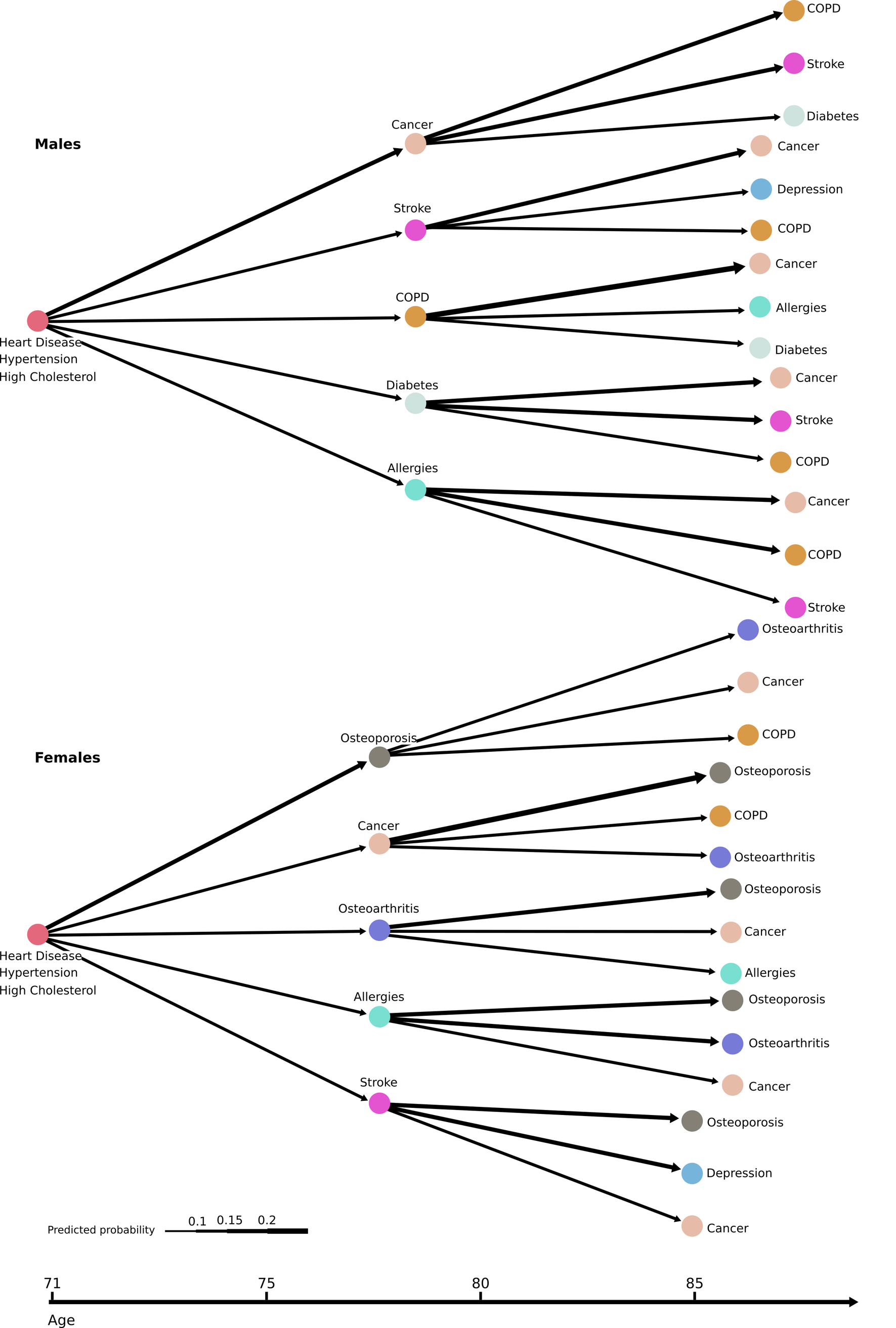

Supplement: S3 Fig — Disease trajectories starting from the most common triad of diseases for males (top) and females (bottom). Trajectories are constructed for retired individuals with no education, and calendar time and age set at the mean levels at t = 0 (70.09 years of age and 2003.37 year time, respectively). The length of the arrows corresponds to the modelled diagnosis postponement time. The width of the arrows corresponds to the modelled probability of obtaining the diagnosis next. (TIF) [file pone.0284496.s003.tif]

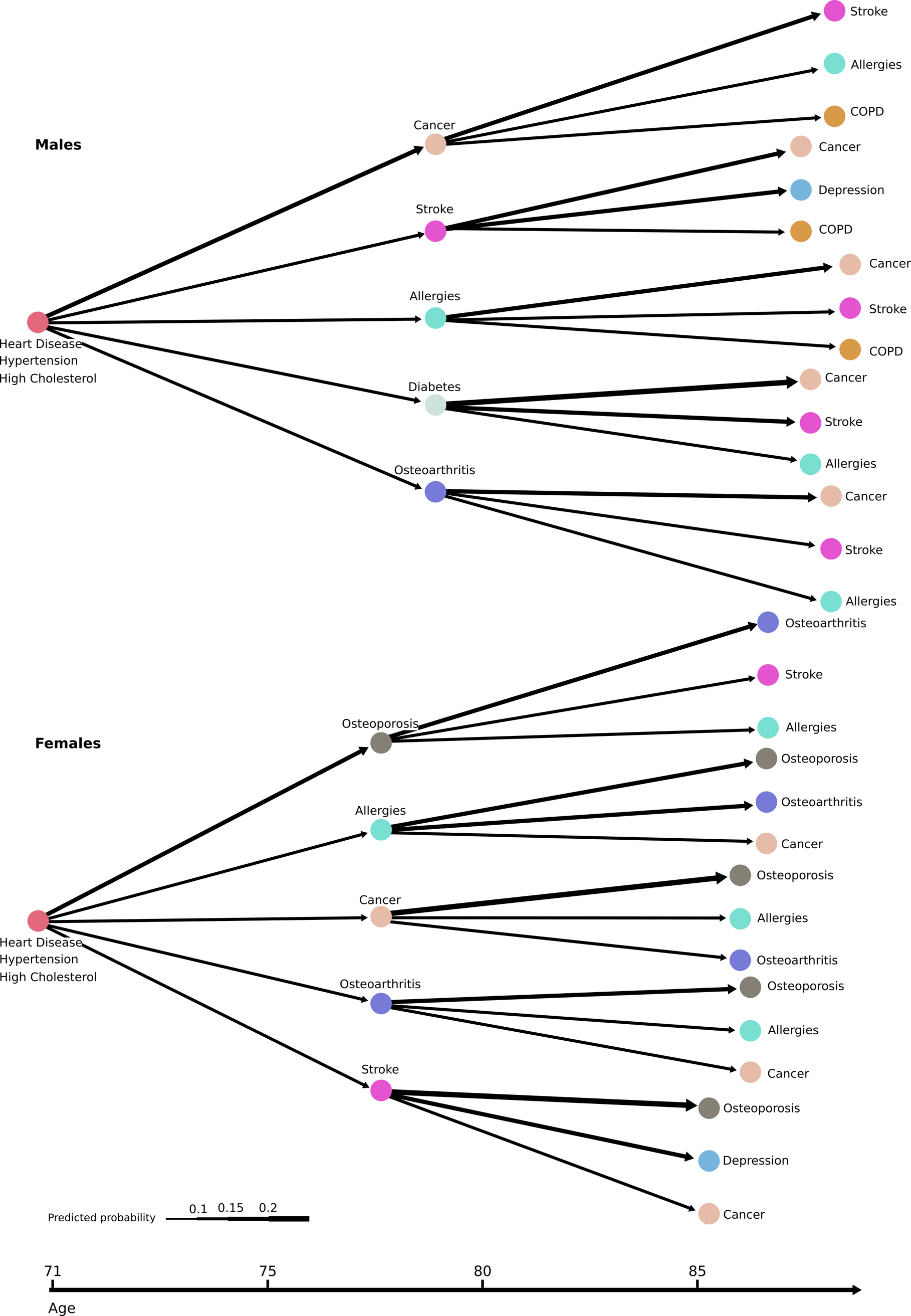

Supplement: S4 Fig — Disease trajectories starting from the most common triad of diseases for males (top) and females (bottom). Trajectories are constructed for retired individuals with no education, and calendar time and age set at the mean levels at t = 0 (70.09 years of age and 2003.37 year time, respectively). The length of the arrows corresponds to the modelled diagnosis postponement time. The width of the arrows corresponds to the modelled probability of obtaining the diagnosis next. (TIF) [file pone.0284496.s004.tif]

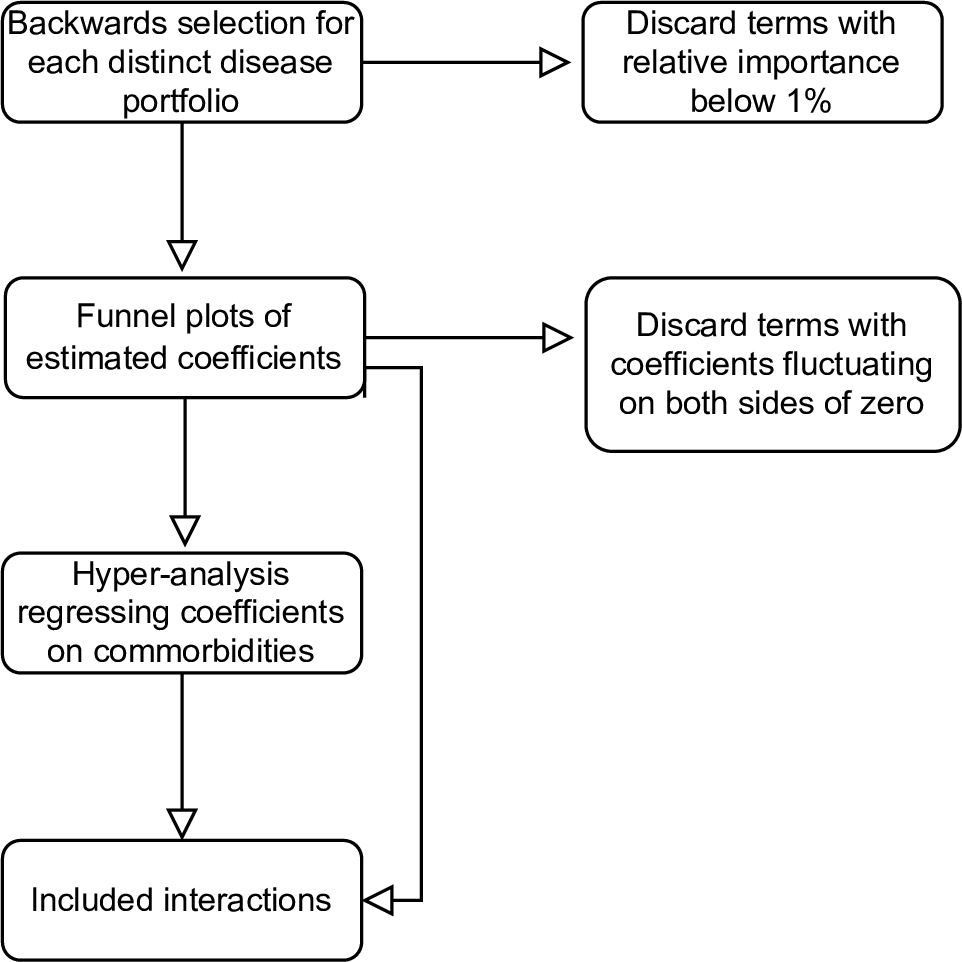

Supplement: S5 Fig — (TIF) [file pone.0284496.s005.tif]
