# Supplementary material for: Longitudinal models for the progression of disease portfolios in a nationwide chronic heart disease population
Source: PLoS One. 2023 Apr 20;18(4):e0284496. doi: 10.1371/journal.pone.0284496 (PMC10118194; doi:10.1371/journal.pone.0284496)
Supplement: S2 Table — (DOCX) [file pone.0284496.s007.docx]

**Table S2: Parameter estimates for effects on the postponement time until a new chronic disease diagnosis.**

|  | Estimate | Std. Error | z value |
| --- | --- | --- | --- |
| (Intercept) | 1.2274 | 0.0054 | 228.4808 |
| Sex Female | -0.0463 | 0.0047 | -9.7776 |
| Age | -0.0133 | 0.0003 | -41.1916 |
| Education Short | 0.0260 | 0.0058 | 4.4680 |
| Education Medium | 0.0881 | 0.0104 | 8.5001 |
| Education Long | 0.1233 | 0.0118 | 10.4720 |
| Education Missing | -0.0016 | 0.0162 | -0.0978 |
| Education Missing pre 1920 | -0.0655 | 0.0125 | -5.2393 |
| Calendar time | -0.0046 | 0.0006 | -7.6919 |
| Occupation Employed | 0.1085 | 0.0053 | 20.6093 |
| Occupation Early retirement pension | -0.0273 | 0.0078 | -3.4979 |
| Occupation Missing | 0.2415 | 0.1133 | 2.1318 |
| Occupation Other | 0.1002 | 0.0174 | 5.7551 |
| Occupation Sick leave, etc. | -0.0383 | 0.0155 | -2.4780 |
| Occupation Student | -0.0459 | 0.0639 | -0.7181 |
| Occupation Unemployed | 0.1688 | 0.0248 | 6.7938 |
| Age^2 | 0.0002 | 0.0000 | 14.9066 |
| Calendar time^2 | -0.0008 | 0.0000 | -21.0542 |
| Stroke | -0.2139 | 0.0074 | -29.0189 |
| Hypertension | 0.1662 | 0.0053 | 31.0754 |
| High cholesterol | 0.3936 | 0.0060 | 65.4401 |
| Allergies | 0.0200 | 0.0053 | 3.7821 |
| JointDisease | -0.2240 | 0.0076 | -29.3054 |
| Osteoporosis | -0.0955 | 0.0062 | -15.5156 |
| Osteoarthritis | -0.0132 | 0.0051 | -2.5965 |
| Back pain | -0.1753 | 0.0046 | -37.9533 |
| COPD | -0.0892 | 0.0054 | -16.4256 |
| Dementia | -0.0230 | 0.0108 | -2.1381 |
| Schizophrenia | -0.0474 | 0.0152 | -3.1225 |
| Depression | -0.0840 | 0.0047 | -18.0628 |
| Diabetes | -0.2153 | 0.0059 | -36.7292 |
| Sex Female:Calendar time | 0.0057 | 0.0004 | 13.9489 |
| Age:Occupation Employed | -0.0027 | 0.0005 | -5.9029 |
| Age:Occupation Early retirement pension | 0.0044 | 0.0006 | 7.1303 |
| Age:Occupation Missing | 0.0020 | 0.0062 | 0.3283 |
| Age:Occupation Other | 0.0020 | 0.0011 | 1.7766 |
| Age:Occupation Sick leave, etc. | 0.0034 | 0.0008 | 4.0718 |
| Age:Occupation Student | -0.0098 | 0.0020 | -4.9293 |
| Age:Occupation Unemployed | 0.0052 | 0.0013 | 3.9022 |
| Age:Education Short | -0.0032 | 0.0002 | -13.1752 |
| Age:Education Medium | -0.0051 | 0.0004 | -12.0489 |
| Age:Education Long | -0.0078 | 0.0005 | -15.1077 |
| Age:Education Missing | 0.0002 | 0.0006 | 0.3077 |
| Age:Education Missing pre 1920 | 0.0035 | 0.0007 | 5.2279 |
| Education Short:Calendar time | 0.0075 | 0.0005 | 15.5460 |
| Education Medium:Calendar time | 0.0118 | 0.0008 | 14.0524 |
| Education Long:Calendar time | 0.0145 | 0.0010 | 14.4867 |
| Education Missing:Calendar time | -0.0007 | 0.0014 | -0.5353 |
| Education Missing pre 1920:Calendar time | -0.0008 | 0.0009 | -0.9007 |
| Calendar time:Occupation Employed | 0.0024 | 0.0006 | 4.1779 |
| Calendar time:Occupation Early retirement pension | 0.0020 | 0.0007 | 2.9443 |
| Calendar time:Occupation Missing | -0.0322 | 0.0161 | -1.9973 |
| Calendar time:Occupation Other | -0.0081 | 0.0018 | -4.5098 |
| Calendar time:Occupation Sick leave, etc. | -0.0127 | 0.0013 | -9.8903 |
| Calendar time:Occupation Student | -0.0011 | 0.0056 | -0.1896 |
| Calendar time:Occupation Unemployed | -0.0088 | 0.0018 | -4.7751 |
| Stroke:Dementia | 0.2660 | 0.0132 | 20.1066 |
| Stroke:Diabetes | 0.0739 | 0.0073 | 10.1638 |
| Hypertension:High cholesterol | 0.0483 | 0.0053 | 9.0685 |
| Hypertension:Schizophrenia | -0.0433 | 0.0159 | -2.7175 |
| High cholesterol:Allergies | -0.0489 | 0.0050 | -9.8158 |
| High cholesterol:Diabetes | 0.2375 | 0.0061 | 38.7031 |
| Osteoporosis:COPD | 0.1334 | 0.0075 | 17.7641 |
| Back pain:Dementia | 0.1265 | 0.0190 | 6.6509 |
| COPD:Schizophrenia | 0.0752 | 0.0165 | 4.5636 |
| Dementia:Schizophrenia | 0.4671 | 0.0196 | 23.8671 |
| Schizophrenia:Diabetes | 0.0510 | 0.0164 | 3.1008 |
| Stroke:High cholesterol | 0.0541 | 0.0064 | 8.4361 |
| Stroke:Hypertension | 0.0630 | 0.0074 | 8.4618 |
| High cholesterol:Dementia | -0.2026 | 0.0127 | -15.9344 |
| Schizophrenia:Depression | -0.0509 | 0.0140 | -3.6418 |
| COPD:Depression | 0.0938 | 0.0069 | 13.6824 |
| Osteoporosis:Back pain | 0.1117 | 0.0088 | 12.7274 |
| Osteoarthritis:Back pain | 0.1231 | 0.0085 | 14.4046 |
| JointDisease:Back pain | 0.0659 | 0.0160 | 4.1155 |
| JointDisease:Osteoarthritis | 0.0564 | 0.0138 | 4.0963 |
| JointDisease:Osteoporosis | 0.0999 | 0.0142 | 7.0392 |
| COPD:Diabetes | 0.0449 | 0.0071 | 6.3159 |
| Sex Female:Hypertension | 0.0524 | 0.0047 | 11.0330 |
| Sex Female:High cholesterol | -0.1159 | 0.0045 | -25.7122 |
| Sex Female:Allergies | 0.0339 | 0.0048 | 7.0119 |
| Sex Female:Osteoporosis | 0.1755 | 0.0069 | 25.4360 |
| Sex Female:Osteoarthritis | 0.0438 | 0.0065 | 6.7205 |
| Sex Female:COPD | -0.0454 | 0.0057 | -7.9291 |
| Sex Female:Dementia | 0.1434 | 0.0117 | 12.2645 |
| Sex Female:Depression | 0.0554 | 0.0057 | 9.7493 |
| Age:Stroke | 0.0060 | 0.0003 | 21.8031 |
| Age:Hypertension | 0.0043 | 0.0002 | 17.9048 |
| Age:High cholesterol | -0.0038 | 0.0002 | -16.2148 |
| Age:Osteoporosis | 0.0057 | 0.0003 | 19.4061 |
| Age:Back pain | -0.0035 | 0.0003 | -12.2007 |
| Age:Schizophrenia | -0.0055 | 0.0005 | -10.7631 |
| Age:Diabetes | 0.0038 | 0.0003 | 12.4630 |
| Education Short:Hypertension | -0.0066 | 0.0058 | -1.1420 |
| Education Medium:Hypertension | -0.0154 | 0.0102 | -1.5092 |
| Education Long:Hypertension | 0.0129 | 0.0120 | 1.0776 |
| Education Missing:Hypertension | 0.0188 | 0.0160 | 1.1751 |
| Education Missing pre 1920:Hypertension | 0.0968 | 0.0079 | 12.1947 |
| Education Short:High cholesterol | -0.0037 | 0.0052 | -0.7066 |
| Education Medium:High cholesterol | -0.0090 | 0.0092 | -0.9796 |
| Education Long:High cholesterol | -0.0233 | 0.0110 | -2.1237 |
| Education Missing:High cholesterol | -0.0525 | 0.0149 | -3.5230 |
| Education Missing pre 1920:High cholesterol | -0.1428 | 0.0112 | -12.7879 |
| Education Short:Allergies | 0.0302 | 0.0056 | 5.3845 |
| Education Medium:Allergies | 0.0315 | 0.0100 | 3.1396 |
| Education Long:Allergies | 0.0684 | 0.0118 | 5.7875 |
| Education Missing:Allergies | 0.0362 | 0.0162 | 2.2284 |
| Education Missing pre 1920:Allergies | 0.0283 | 0.0071 | 3.9771 |
| Education Short:COPD | -0.0178 | 0.0064 | -2.7733 |
| Education Medium:COPD | -0.0150 | 0.0128 | -1.1699 |
| Education Long:COPD | -0.0824 | 0.0165 | -4.9960 |
| Education Missing:COPD | 0.0218 | 0.0194 | 1.1274 |
| Education Missing pre 1920:COPD | 0.0262 | 0.0089 | 2.9258 |
| Education Short:Diabetes | -0.0283 | 0.0066 | -4.3094 |
| Education Medium:Diabetes | -0.0596 | 0.0125 | -4.7594 |
| Education Long:Diabetes | -0.0898 | 0.0155 | -5.8108 |
| Education Missing:Diabetes | 0.0455 | 0.0168 | 2.7176 |
| Education Missing pre 1920:Diabetes | 0.0981 | 0.0100 | 9.8261 |
| Calendar time:Stroke | -0.0056 | 0.0006 | -9.6836 |
| Calendar time:Hypertension | -0.0096 | 0.0005 | -19.7847 |
| Calendar time:High cholesterol | 0.0103 | 0.0004 | 23.4496 |
| Calendar time:Osteoarthritis | 0.0073 | 0.0006 | 12.2476 |
| Calendar time:Back pain | 0.0081 | 0.0006 | 13.1318 |
| Calendar time:Diabetes | -0.0021 | 0.0006 | -3.6043 |
