# Supplementary material for: Longitudinal models for the progression of disease portfolios in a nationwide chronic heart disease population
Source: PLoS One. 2023 Apr 20;18(4):e0284496. doi: 10.1371/journal.pone.0284496 (PMC10118194; doi:10.1371/journal.pone.0284496)
Supplement: S3 Table — (DOCX) [file pone.0284496.s008.docx]

**Table S3: Parameter estimates for effects on obtaining stroke as the next chronic disease diagnosis.**

|  | Estimate | Std. Error | z value |
| --- | --- | --- | --- |
| (Intercept) | -2.7030 | 0.0196 | -138.15 |
| Sex Female | -0.3253 | 0.0190 | -17.11 |
| Age | 0.0421 | 0.0010 | 42.61 |
| Education Short | 0.0072 | 0.0215 | 0.33 |
| Education Medium | -0.0160 | 0.0390 | -0.41 |
| Education Long | 0.0041 | 0.0438 | 0.09 |
| Education Missing | -0.0179 | 0.0619 | -0.29 |
| Education Missing pre 1920 | 0.5739 | 0.0377 | 15.23 |
| Calendar time | -0.0459 | 0.0022 | -20.58 |
| Occupation Employed | -0.0316 | 0.0184 | -1.71 |
| Occupation Early retirement pension | 0.0390 | 0.0300 | 1.30 |
| Occupation Missing | -0.6440 | 0.5572 | -1.16 |
| Occupation Other | 0.0628 | 0.0660 | 0.95 |
| Occupation Sick leave, etc. | -0.0500 | 0.0621 | -0.81 |
| Occupation Student | -0.7081 | 0.3142 | -2.25 |
| Occupation Unemployed | -0.2040 | 0.1093 | -1.87 |
| Age^2 | 0.0003 | 0.0000 | 7.40 |
| Calendar time^2 | 0.0009 | 0.0001 | 6.57 |
| Calendar time^3 | 0.0001 | 0.0000 | 5.86 |
| Hypertension | 0.3895 | 0.0194 | 20.06 |
| High cholesterol | 0.2315 | 0.0202 | 11.44 |
| Allergies | 0.0276 | 0.0082 | 3.36 |
| JointDisease | -0.0483 | 0.0240 | -2.01 |
| Osteoporosis | 0.1404 | 0.0201 | 6.98 |
| Osteoarthritis | 0.0195 | 0.0124 | 1.57 |
| Back pain | -0.0361 | 0.0155 | -2.34 |
| Cancer | 0.1061 | 0.0120 | 8.87 |
| COPD | -0.0747 | 0.0119 | -6.27 |
| Dementia | 0.3944 | 0.0216 | 18.26 |
| Schizophrenia | 0.0185 | 0.0294 | 0.63 |
| Depression | 0.1230 | 0.0261 | 4.72 |
| Diabetes | 0.2005 | 0.0146 | 13.69 |
| Sex Female:Calendar time | 0.0061 | 0.0014 | 4.20 |
| Age:Occupation Employed | -0.0050 | 0.0016 | -3.07 |
| Age:Occupation Early retirement pension | -0.0095 | 0.0024 | -4.04 |
| Age:Occupation Missing | -0.0235 | 0.0297 | -0.79 |
| Age:Occupation Other | -0.0073 | 0.0046 | -1.56 |
| Age:Occupation Sick leave, etc. | -0.0098 | 0.0034 | -2.84 |
| Age:Occupation Student | -0.0322 | 0.0092 | -3.50 |
| Age:Occupation Unemployed | -0.0136 | 0.0060 | -2.24 |
| Age:Education Short | -0.0006 | 0.0009 | -0.60 |
| Age:Education Medium | 0.0035 | 0.0016 | 2.15 |
| Age:Education Long | 0.0031 | 0.0019 | 1.61 |
| Age:Education Missing | 0.0003 | 0.0026 | 0.11 |
| Age:Education Missing pre 1920 | -0.0435 | 0.0022 | -20.05 |
| Education Short:Calendar time | 0.0010 | 0.0017 | 0.55 |
| Education Medium:Calendar time | 0.0047 | 0.0031 | 1.53 |
| Education Long:Calendar time | 0.0081 | 0.0036 | 2.29 |
| Education Missing:Calendar time | 0.0069 | 0.0051 | 1.36 |
| Education Missing pre 1920:Calendar time | 0.0331 | 0.0026 | 12.51 |
| Calendar time:Occupation Employed | 0.0097 | 0.0023 | 4.28 |
| Calendar time:Occupation Early retirement pension | 0.0119 | 0.0026 | 4.52 |
| Calendar time:Occupation Missing | -0.0624 | 0.0835 | -0.75 |
| Calendar time:Occupation Other | 0.0266 | 0.0069 | 3.86 |
| Calendar time:Occupation Sick leave, etc. | 0.0062 | 0.0054 | 1.14 |
| Calendar time:Occupation Student | 0.0498 | 0.0273 | 1.83 |
| Calendar time:Occupation Unemployed | -0.0094 | 0.0082 | -1.15 |
| Hypertension:High cholesterol | 0.0920 | 0.0209 | 4.39 |
| Hypertension:Depression | -0.0761 | 0.0276 | -2.76 |
| High cholesterol:Diabetes | 0.0860 | 0.0189 | 4.56 |
| Osteoporosis:COPD | 0.0827 | 0.0242 | 3.41 |
| COPD:Schizophrenia | 0.2388 | 0.0547 | 4.36 |
| Dementia:Schizophrenia | 0.1831 | 0.0562 | 3.26 |
| High cholesterol:Dementia | 0.2034 | 0.0373 | 5.45 |
| COPD:Depression | 0.2067 | 0.0234 | 8.82 |
| Osteoporosis:Back pain | 0.0861 | 0.0279 | 3.08 |
| Osteoarthritis:Back pain | 0.1196 | 0.0290 | 4.12 |
| JointDisease:Osteoporosis | 0.1573 | 0.0459 | 3.43 |
| Education Short:Hypertension | 0.0426 | 0.0230 | 1.86 |
| Education Medium:Hypertension | 0.1003 | 0.0419 | 2.40 |
| Education Long:Hypertension | 0.1185 | 0.0479 | 2.48 |
| Education Missing:Hypertension | 0.0974 | 0.0656 | 1.48 |
| Education Missing pre 1920:Hypertension | 0.0848 | 0.0243 | 3.50 |
| Sex Female:Hypertension | 0.1594 | 0.0191 | 8.35 |
| Sex Female:High cholesterol | -0.0975 | 0.0156 | -6.25 |
| Age:High cholesterol | -0.0101 | 0.0008 | -13.11 |
| Sex Female:Osteoporosis | 0.1292 | 0.0220 | 5.86 |
| Age:COPD | -0.0066 | 0.0009 | -7.03 |
| Calendar time:Diabetes | -0.0117 | 0.0018 | -6.54 |
