# Supplementary material for: Longitudinal models for the progression of disease portfolios in a nationwide chronic heart disease population
Source: PLoS One. 2023 Apr 20;18(4):e0284496. doi: 10.1371/journal.pone.0284496 (PMC10118194; doi:10.1371/journal.pone.0284496)
Supplement: S4 Table — (DOCX) [file pone.0284496.s009.docx]

**Table S4: Parameter estimates for effects on obtaining hypertension as the next chronic disease diagnosis.**

|  | Estimate | Std. Error | z value |
| --- | --- | --- | --- |
| (Intercept) | -1.0893 | 0.0139 | -78.16 |
| Sex Female | 0.1529 | 0.0113 | 13.57 |
| Age | 0.0119 | 0.0009 | 12.59 |
| Education Short | 0.0439 | 0.0115 | 3.82 |
| Education Medium | 0.0362 | 0.0190 | 1.91 |
| Education Long | 0.0969 | 0.0220 | 4.40 |
| Education Missing | 0.0620 | 0.0302 | 2.05 |
| Education Missing pre 1920 | -0.1377 | 0.0284 | -4.84 |
| Calendar time | -0.0116 | 0.0027 | -4.24 |
| Occupation Employed | 0.0661 | 0.0190 | 3.47 |
| Occupation Early retirement pension | -0.0769 | 0.0283 | -2.72 |
| Occupation Missing | 0.7649 | 0.3163 | 2.42 |
| Occupation Other | 0.0625 | 0.0627 | 1.00 |
| Occupation Sick leave, etc. | 0.0852 | 0.0583 | 1.46 |
| Occupation Student | -0.3595 | 0.2023 | -1.78 |
| Occupation Unemployed | 0.2270 | 0.0831 | 2.73 |
| Calendar time^2 | 0.0023 | 0.0002 | 12.83 |
| Calendar time^3 | -0.0005 | 0.0000 | -16.87 |
| Stroke | -0.0289 | 0.0186 | -1.55 |
| High cholesterol | 0.2295 | 0.0130 | 17.61 |
| Allergies | 0.1182 | 0.0107 | 11.08 |
| JointDisease | -0.2203 | 0.0361 | -6.10 |
| Osteoporosis | -0.0313 | 0.0176 | -1.78 |
| Osteoarthritis | 0.0927 | 0.0182 | 5.09 |
| Back pain | -0.0806 | 0.0189 | -4.27 |
| Cancer | 0.1293 | 0.0161 | 8.02 |
| COPD | 0.0077 | 0.0173 | 0.45 |
| Dementia | -0.2343 | 0.0346 | -6.77 |
| Schizophrenia | -0.0091 | 0.0328 | -0.28 |
| Depression | -0.0904 | 0.0151 | -5.98 |
| Diabetes | 0.1385 | 0.0223 | 6.22 |
| Sex Female:Calendar time | -0.0070 | 0.0017 | -4.11 |
| Age:Occupation Employed | 0.0015 | 0.0013 | 1.19 |
| Age:Occupation Early retirement pension | -0.0041 | 0.0019 | -2.13 |
| Age:Occupation Missing | 0.0030 | 0.0161 | 0.19 |
| Age:Occupation Other | -0.0020 | 0.0037 | -0.55 |
| Age:Occupation Sick leave, etc. | 0.0057 | 0.0027 | 2.12 |
| Age:Occupation Student | -0.0097 | 0.0055 | -1.76 |
| Age:Occupation Unemployed | 0.0129 | 0.0042 | 3.05 |
| Education Short:Calendar time | 0.0018 | 0.0020 | 0.88 |
| Education Medium:Calendar time | 0.0009 | 0.0034 | 0.28 |
| Education Long:Calendar time | -0.0012 | 0.0039 | -0.32 |
| Education Missing:Calendar time | -0.0043 | 0.0055 | -0.79 |
| Education Missing pre 1920:Calendar time | -0.0271 | 0.0037 | -7.24 |
| High cholesterol:Diabetes | 0.4481 | 0.0279 | 16.04 |
| Osteoporosis:COPD | 0.1908 | 0.0368 | 5.19 |
| Back pain:Dementia | 0.3386 | 0.1027 | 3.30 |
| Dementia:Schizophrenia | 0.4324 | 0.0832 | 5.19 |
| COPD:Depression | 0.1435 | 0.0339 | 4.24 |
| Osteoporosis:Back pain | 0.1529 | 0.0432 | 3.54 |
| Osteoarthritis:Back pain | 0.1336 | 0.0439 | 3.04 |
| JointDisease:Back pain | 0.2808 | 0.0956 | 2.94 |
| Stroke:Dementia | 0.2604 | 0.0657 | 3.96 |
| Stroke:High cholesterol | 0.2162 | 0.0289 | 7.49 |
| Sex Female:COPD | -0.1161 | 0.0262 | -4.44 |
| Sex Female:Diabetes | -0.1841 | 0.0294 | -6.27 |
| Age:High cholesterol | -0.0149 | 0.0008 | -18.23 |
| Calendar time:High cholesterol | 0.0294 | 0.0019 | 15.46 |
| Calendar time:Osteoarthritis | 0.0118 | 0.0030 | 3.92 |
