# Supplementary material for: Longitudinal models for the progression of disease portfolios in a nationwide chronic heart disease population
Source: PLoS One. 2023 Apr 20;18(4):e0284496. doi: 10.1371/journal.pone.0284496 (PMC10118194; doi:10.1371/journal.pone.0284496)
Supplement: S5 Table — (DOCX) [file pone.0284496.s010.docx]

**Table S5: Parameter estimates for effects on obtaining high cholesterol as the next chronic disease diagnosis.**

|  | Estimate | Std. Error | z value |
| --- | --- | --- | --- |
| (Intercept) | -0.9630 | 0.0125 | -76.77 |
| Sex Female | -0.5408 | 0.0150 | -36.11 |
| Age | -0.0818 | 0.0009 | -89.32 |
| Education Short | 0.0069 | 0.0104 | 0.66 |
| Education Medium | 0.0265 | 0.0188 | 1.41 |
| Education Long | -0.0281 | 0.0214 | -1.32 |
| Education Missing | -0.0735 | 0.0302 | -2.44 |
| Education Missing pre 1920 | 1.7220 | 0.0765 | 22.50 |
| Calendar time | 0.0419 | 0.0021 | 20.19 |
| Occupation Employed | 0.1760 | 0.0169 | 10.40 |
| Occupation Early retirement pension | -0.4441 | 0.0245 | -18.13 |
| Occupation Missing | -0.0031 | 0.3438 | -0.01 |
| Occupation Other | 0.1156 | 0.0524 | 2.21 |
| Occupation Sick leave, etc. | 0.0527 | 0.0490 | 1.08 |
| Occupation Student | -0.3931 | 0.2068 | -1.90 |
| Occupation Unemployed | 0.1898 | 0.0730 | 2.60 |
| Age^2 | -0.0015 | 0.0000 | -39.27 |
| Calendar time^2 | -0.0049 | 0.0001 | -37.00 |
| Calendar time^3 | 0.0006 | 0.0000 | 25.64 |
| Stroke | 0.2979 | 0.0190 | 15.65 |
| Hypertension | 0.3936 | 0.0100 | 39.26 |
| Allergies | 0.0466 | 0.0092 | 5.09 |
| JointDisease | -0.3478 | 0.0251 | -13.87 |
| Osteoporosis | -0.5529 | 0.0229 | -24.18 |
| Osteoarthritis | -0.0091 | 0.0133 | -0.68 |
| Back pain | -0.0980 | 0.0141 | -6.96 |
| Cancer | -0.3082 | 0.0149 | -20.74 |
| COPD | -0.2845 | 0.0126 | -22.66 |
| Dementia | -0.9651 | 0.0397 | -24.32 |
| Schizophrenia | -0.5255 | 0.0560 | -9.39 |
| Depression | -0.2385 | 0.0166 | -14.37 |
| Diabetes | 0.4910 | 0.0159 | 30.84 |
| Age:Occupation Employed | 0.0243 | 0.0016 | 15.53 |
| Age:Occupation Early retirement pension | 0.0129 | 0.0020 | 6.57 |
| Age:Occupation Missing | -0.0003 | 0.0187 | -0.01 |
| Age:Occupation Other | 0.0294 | 0.0035 | 8.49 |
| Age:Occupation Sick leave, etc. | 0.0303 | 0.0027 | 11.06 |
| Age:Occupation Student | 0.0077 | 0.0070 | 1.10 |
| Age:Occupation Unemployed | 0.0304 | 0.0040 | 7.60 |
| Age:Education Short | 0.0047 | 0.0008 | 6.04 |
| Age:Education Medium | 0.0078 | 0.0013 | 5.90 |
| Age:Education Long | 0.0102 | 0.0016 | 6.25 |
| Age:Education Missing | 0.0014 | 0.0020 | 0.72 |
| Age:Education Missing pre 1920 | -0.1234 | 0.0051 | -24.30 |
| Education Short:Calendar time | -0.0127 | 0.0016 | -8.21 |
| Education Medium:Calendar time | -0.0104 | 0.0027 | -3.91 |
| Education Long:Calendar time | -0.0040 | 0.0031 | -1.28 |
| Education Missing:Calendar time | -0.0045 | 0.0043 | -1.04 |
| Education Missing pre 1920:Calendar time | 0.2220 | 0.0056 | 39.50 |
| Calendar time:Occupation Employed | -0.0626 | 0.0018 | -35.12 |
| Calendar time:Occupation Early retirement pension | -0.0379 | 0.0022 | -17.15 |
| Calendar time:Occupation Missing | 0.0200 | 0.0418 | 0.48 |
| Calendar time:Occupation Other | -0.0487 | 0.0053 | -9.25 |
| Calendar time:Occupation Sick leave, etc. | -0.0644 | 0.0040 | -16.24 |
| Calendar time:Occupation Student | -0.0729 | 0.0170 | -4.30 |
| Calendar time:Occupation Unemployed | -0.0426 | 0.0055 | -7.76 |
| Osteoporosis:COPD | 0.1848 | 0.0269 | 6.87 |
| Osteoarthritis:Dementia | 0.3681 | 0.0740 | 4.97 |
| Back pain:Dementia | 0.2455 | 0.0843 | 2.91 |
| COPD:Schizophrenia | 0.1726 | 0.0588 | 2.94 |
| Dementia:Schizophrenia | 0.3431 | 0.0852 | 4.02 |
| Schizophrenia:Diabetes | 0.2133 | 0.0570 | 3.74 |
| Schizophrenia:Depression | 0.2958 | 0.0516 | 5.73 |
| Cancer:Depression | 0.1511 | 0.0337 | 4.48 |
| COPD:Depression | 0.1264 | 0.0243 | 5.21 |
| Osteoarthritis:Back pain | 0.1051 | 0.0313 | 3.36 |
| JointDisease:Osteoporosis | 0.2162 | 0.0532 | 4.07 |
| COPD:Diabetes | 0.1893 | 0.0250 | 7.58 |
| Stroke:Dementia | 0.5209 | 0.0558 | 9.34 |
| Stroke:Diabetes | -0.3148 | 0.0261 | -12.07 |
| Hypertension:Schizophrenia | -0.1717 | 0.0584 | -2.94 |
| Sex Female:Stroke | 0.1959 | 0.0204 | 9.60 |
| Sex Female:Hypertension | 0.2507 | 0.0165 | 15.15 |
| Sex Female:Osteoporosis | 0.3991 | 0.0256 | 15.59 |
| Sex Female:Depression | 0.1173 | 0.0201 | 5.83 |
| Age:Allergies | 0.0074 | 0.0007 | 10.53 |
| Age:Osteoarthritis | 0.0061 | 0.0011 | 5.39 |
| Age:Cancer | 0.0137 | 0.0014 | 10.02 |
| Education Short:Stroke | 0.0611 | 0.0233 | 2.62 |
| Education Medium:Stroke | 0.1092 | 0.0448 | 2.44 |
| Education Long:Stroke | 0.1440 | 0.0508 | 2.84 |
| Education Missing:Stroke | 0.1871 | 0.0692 | 2.70 |
| Education Missing pre 1920:Stroke | 0.2463 | 0.0339 | 7.27 |
| Calendar time:Allergies | -0.0109 | 0.0016 | -7.00 |
| Calendar time:Osteoarthritis | -0.0104 | 0.0022 | -4.65 |
| Calendar time:Back pain | -0.0062 | 0.0023 | -2.73 |
| Calendar time:Cancer | -0.0087 | 0.0024 | -3.55 |
| Calendar time:COPD | 0.0124 | 0.0019 | 6.70 |
| Sex Female:Diabetes | 0.2477 | 0.0190 | 13.01 |
| Age:Diabetes | -0.0132 | 0.0009 | -14.84 |
| Calendar time:Diabetes | 0.0113 | 0.0019 | 5.85 |
