# Supplementary material for: Longitudinal models for the progression of disease portfolios in a nationwide chronic heart disease population
Source: PLoS One. 2023 Apr 20;18(4):e0284496. doi: 10.1371/journal.pone.0284496 (PMC10118194; doi:10.1371/journal.pone.0284496)
Supplement: S6 Table — (DOCX) [file pone.0284496.s011.docx]

**Table S6: Parameter estimates for effects on obtaining allergies as the next chronic disease diagnosis.**

|  | Estimate | Std. Error | z value |
| --- | --- | --- | --- |
| (Intercept) | -3.0804 | 0.0154 | -200.60 |
| Sex Female | 0.2475 | 0.0106 | 23.36 |
| Age | 0.0044 | 0.0009 | 4.68 |
| Education Short | 0.1209 | 0.0120 | 10.06 |
| Education Medium | 0.1731 | 0.0218 | 7.95 |
| Education Long | 0.3496 | 0.0241 | 14.53 |
| Education Missing | 0.2776 | 0.0339 | 8.19 |
| Education Missing pre 1920 | 0.1232 | 0.0360 | 3.42 |
| Calendar time | -0.0499 | 0.0026 | -19.50 |
| Occupation Employed | 0.1523 | 0.0151 | 10.06 |
| Occupation Early retirement pension | 0.1910 | 0.0175 | 10.89 |
| Occupation Missing | -0.1960 | 0.3335 | -0.59 |
| Occupation Other | 0.0805 | 0.0384 | 2.09 |
| Occupation Sick leave, etc. | 0.1087 | 0.0278 | 3.91 |
| Occupation Student | 0.4259 | 0.0894 | 4.76 |
| Occupation Unemployed | 0.1813 | 0.0390 | 4.65 |
| Age^2 | 0.0005 | 0.0000 | 17.93 |
| Calendar time^2 | 0.0021 | 0.0002 | 13.54 |
| Calendar time^3 | -0.0001 | 0.0000 | -4.29 |
| Stroke | -0.2137 | 0.0359 | -5.95 |
| Hypertension | 0.3865 | 0.0094 | 40.97 |
| High cholesterol | 0.3345 | 0.0136 | 24.55 |
| Osteoporosis | 0.0205 | 0.0145 | 1.41 |
| Osteoarthritis | 0.0608 | 0.0144 | 4.22 |
| Back pain | 0.0326 | 0.0151 | 2.16 |
| Cancer | 0.1669 | 0.0187 | 8.94 |
| COPD | 0.3312 | 0.0146 | 22.65 |
| Dementia | 0.0037 | 0.0303 | 0.12 |
| Schizophrenia | 0.0246 | 0.0352 | 0.70 |
| Depression | 0.1924 | 0.0114 | 16.85 |
| Diabetes | -0.1466 | 0.0150 | -9.75 |
| Age:Education Short | -0.0009 | 0.0009 | -1.01 |
| Age:Education Medium | -0.0073 | 0.0015 | -4.88 |
| Age:Education Long | -0.0097 | 0.0018 | -5.46 |
| Age:Education Missing | 0.0009 | 0.0021 | 0.44 |
| Age:Education Missing pre 1920 | -0.0093 | 0.0023 | -4.15 |
| Education Short:Calendar time | 0.0047 | 0.0018 | 2.63 |
| Education Medium:Calendar time | 0.0114 | 0.0030 | 3.74 |
| Education Long:Calendar time | 0.0116 | 0.0035 | 3.30 |
| Education Missing:Calendar time | -0.0031 | 0.0049 | -0.63 |
| Education Missing pre 1920:Calendar time | -0.0038 | 0.0032 | -1.16 |
| Calendar time:Occupation Employed | 0.0168 | 0.0021 | 7.94 |
| Calendar time:Occupation Early retirement pension | 0.0105 | 0.0025 | 4.23 |
| Calendar time:Occupation Missing | 0.1031 | 0.0632 | 1.63 |
| Calendar time:Occupation Other | -0.0007 | 0.0065 | -0.11 |
| Calendar time:Occupation Sick leave, etc. | 0.0111 | 0.0045 | 2.44 |
| Calendar time:Occupation Student | 0.0187 | 0.0160 | 1.16 |
| Calendar time:Occupation Unemployed | 0.0188 | 0.0064 | 2.93 |
| Osteoporosis:COPD | 0.1495 | 0.0274 | 5.46 |
| Osteoarthritis:Dementia | 0.1708 | 0.0621 | 2.75 |
| COPD:Schizophrenia | 0.1959 | 0.0584 | 3.35 |
| Dementia:Schizophrenia | 0.2734 | 0.0658 | 4.15 |
| Schizophrenia:Depression | -0.1497 | 0.0530 | -2.83 |
| Osteoarthritis:Back pain | 0.1235 | 0.0326 | 3.79 |
| High cholesterol:Diabetes | 0.1800 | 0.0202 | 8.93 |
| High cholesterol:Dementia | -0.2173 | 0.0478 | -4.55 |
| Stroke:Dementia | 0.4070 | 0.0470 | 8.65 |
| Stroke:Diabetes | 0.1475 | 0.0269 | 5.48 |
| Stroke:Hypertension | 0.1143 | 0.0312 | 3.66 |
| Stroke:High cholesterol | 0.1034 | 0.0244 | 4.24 |
| Sex Female:Stroke | -0.1254 | 0.0228 | -5.51 |
| Sex Female:High cholesterol | -0.1536 | 0.0157 | -9.76 |
| Sex Female:Cancer | -0.1359 | 0.0266 | -5.12 |
| Sex Female:COPD | -0.0673 | 0.0202 | -3.33 |
| Age:High cholesterol | -0.0041 | 0.0007 | -5.50 |
| Education Short:Stroke | -0.0894 | 0.0271 | -3.30 |
| Education Medium:Stroke | -0.0534 | 0.0505 | -1.06 |
| Education Long:Stroke | -0.2343 | 0.0596 | -3.93 |
| Education Missing:Stroke | -0.1701 | 0.0796 | -2.14 |
| Education Missing pre 1920:Stroke | 0.1594 | 0.0314 | 5.07 |
| Calendar time:High cholesterol | 0.0224 | 0.0016 | 13.85 |
| Calendar time:Osteoporosis | 0.0149 | 0.0022 | 6.90 |
| Calendar time:Osteoarthritis | 0.0126 | 0.0023 | 5.57 |
| Calendar time:Back pain | 0.0179 | 0.0023 | 7.66 |
| Calendar time:Cancer | 0.0088 | 0.0024 | 3.67 |
| Calendar time:COPD | 0.0079 | 0.0019 | 4.25 |
| Calendar time:Depression | 0.0184 | 0.0019 | 9.45 |
