# Supplementary material for: Longitudinal models for the progression of disease portfolios in a nationwide chronic heart disease population
Source: PLoS One. 2023 Apr 20;18(4):e0284496. doi: 10.1371/journal.pone.0284496 (PMC10118194; doi:10.1371/journal.pone.0284496)
Supplement: S7 Table — (DOCX) [file pone.0284496.s012.docx]

**Table S7: Parameter estimates for effects on obtaining joint disease as the next chronic disease diagnosis.**

|  | Estimate | Std. Error | z value |
| --- | --- | --- | --- |
| (Intercept) | -4.8625 | 0.0322 | -151.13 |
| Sex Female | -0.1834 | 0.0439 | -4.18 |
| Age | 0.0049 | 0.0015 | 3.27 |
| Education Short | -0.0047 | 0.0197 | -0.24 |
| Education Medium | 0.0155 | 0.0362 | 0.43 |
| Education Long | -0.1078 | 0.0432 | -2.49 |
| Education Missing | -0.1522 | 0.0634 | -2.40 |
| Education Missing pre 1920 | 0.3383 | 0.0800 | 4.23 |
| Calendar time | -0.0040 | 0.0039 | -1.03 |
| Occupation Employed | -0.0983 | 0.0284 | -3.46 |
| Occupation Early retirement pension | 0.0547 | 0.0286 | 1.91 |
| Occupation Missing | -9.1201 | 42.6820 | -0.21 |
| Occupation Other | -0.1707 | 0.0826 | -2.07 |
| Occupation Sick leave, etc. | -0.0976 | 0.0595 | -1.64 |
| Occupation Student | 0.2295 | 0.2295 | 1.00 |
| Occupation Unemployed | -0.2202 | 0.0898 | -2.45 |
| Calendar time^2 | 0.0007 | 0.0003 | 2.39 |
| Calendar time^3 | -0.0002 | 0.0001 | -3.56 |
| Stroke | -0.0102 | 0.0360 | -0.28 |
| Hypertension | 0.8482 | 0.0268 | 31.62 |
| High cholesterol | 0.3821 | 0.0228 | 16.76 |
| Allergies | 0.1533 | 0.0163 | 9.39 |
| Osteoporosis | 0.2250 | 0.0214 | 10.52 |
| Osteoarthritis | 0.5685 | 0.0193 | 29.42 |
| Back pain | 0.1162 | 0.0226 | 5.14 |
| COPD | 0.3312 | 0.0209 | 15.83 |
| Dementia | -0.5938 | 0.0651 | -9.12 |
| Schizophrenia | -0.4001 | 0.0591 | -6.77 |
| Depression | -0.1434 | 0.0207 | -6.92 |
| Diabetes | 0.1919 | 0.0291 | 6.59 |
| Age:Education Short | -0.0057 | 0.0018 | -3.22 |
| Age:Education Medium | 0.0039 | 0.0032 | 1.21 |
| Age:Education Long | 0.0024 | 0.0041 | 0.57 |
| Age:Education Missing | -0.0008 | 0.0052 | -0.16 |
| Age:Education Missing pre 1920 | -0.0244 | 0.0047 | -5.24 |
| Education Short:Calendar time | 0.0134 | 0.0033 | 4.09 |
| Education Medium:Calendar time | 0.0126 | 0.0060 | 2.09 |
| Education Long:Calendar time | 0.0136 | 0.0075 | 1.83 |
| Education Missing:Calendar time | 0.0192 | 0.0104 | 1.84 |
| Education Missing pre 1920:Calendar time | 0.0139 | 0.0066 | 2.11 |
| COPD:Diabetes | 0.1569 | 0.0383 | 4.10 |
| High cholesterol:Diabetes | 0.3121 | 0.0352 | 8.87 |
| Stroke:Dementia | 0.4196 | 0.1059 | 3.96 |
| Stroke:High cholesterol | 0.1241 | 0.0404 | 3.07 |
| Sex Female:Hypertension | -0.1970 | 0.0450 | -4.38 |
| Sex Female:High cholesterol | -0.1378 | 0.0303 | -4.55 |
| Sex Female:Stroke | -0.1243 | 0.0413 | -3.01 |
