# Supplementary material for: Longitudinal models for the progression of disease portfolios in a nationwide chronic heart disease population
Source: PLoS One. 2023 Apr 20;18(4):e0284496. doi: 10.1371/journal.pone.0284496 (PMC10118194; doi:10.1371/journal.pone.0284496)
Supplement: S8 Table — (DOCX) [file pone.0284496.s013.docx]

**Table S8: Parameter estimates for effects on obtaining osteoporosis as the next chronic disease diagnosis.**

|  | Estimate | Std. Error | z value |
| --- | --- | --- | --- |
| (Intercept) | -3.2883 | 0.0168 | -195.89 |
| Sex Female | 0.9742 | 0.0116 | 84.28 |
| Age | 0.0419 | 0.0010 | 41.62 |
| Education Short | -0.0019 | 0.0098 | -0.20 |
| Education Medium | 0.0372 | 0.0184 | 2.02 |
| Education Long | 0.0690 | 0.0213 | 3.23 |
| Education Missing | -0.1758 | 0.0319 | -5.51 |
| Education Missing pre 1920 | -0.0678 | 0.0307 | -2.21 |
| Calendar time | 0.0083 | 0.0020 | 4.10 |
| Occupation Employed | -0.1037 | 0.0202 | -5.13 |
| Occupation Early retirement pension | -0.0919 | 0.0302 | -3.04 |
| Occupation Missing | -0.3320 | 0.5336 | -0.62 |
| Occupation Other | -0.1106 | 0.0733 | -1.51 |
| Occupation Sick leave, etc. | -0.1404 | 0.0679 | -2.07 |
| Occupation Student | 0.3360 | 0.3664 | 0.92 |
| Occupation Unemployed | -0.2017 | 0.1299 | -1.55 |
| Age^2 | 0.0001 | 0.0000 | 2.66 |
| Calendar time^2 | -0.0014 | 0.0001 | -10.26 |
| Stroke | 0.2858 | 0.0157 | 18.26 |
| Hypertension | 0.2622 | 0.0132 | 19.89 |
| High cholesterol | 0.1387 | 0.0214 | 6.47 |
| Allergies | 0.1854 | 0.0102 | 18.10 |
| JointDisease | 0.4514 | 0.0192 | 23.56 |
| Osteoarthritis | 0.0454 | 0.0114 | 3.98 |
| Back pain | 0.3647 | 0.0188 | 19.44 |
| Cancer | 0.4730 | 0.0180 | 26.29 |
| COPD | 0.7556 | 0.0147 | 51.48 |
| Dementia | 0.7687 | 0.0186 | 41.42 |
| Schizophrenia | 0.3153 | 0.0289 | 10.93 |
| Depression | 0.4799 | 0.0177 | 27.18 |
| Diabetes | -0.0549 | 0.0201 | -2.73 |
| Age:Occupation Employed | 0.0030 | 0.0017 | 1.74 |
| Age:Occupation Early retirement pension | -0.0118 | 0.0025 | -4.76 |
| Age:Occupation Missing | 0.0063 | 0.0390 | 0.16 |
| Age:Occupation Other | -0.0024 | 0.0053 | -0.45 |
| Age:Occupation Sick leave, etc. | 0.0075 | 0.0038 | 1.95 |
| Age:Occupation Student | 0.0236 | 0.0156 | 1.51 |
| Age:Occupation Unemployed | -0.0012 | 0.0074 | -0.16 |
| Age:Education Short | -0.0051 | 0.0010 | -5.17 |
| Age:Education Medium | -0.0030 | 0.0018 | -1.71 |
| Age:Education Long | -0.0061 | 0.0021 | -2.89 |
| Age:Education Missing | -0.0024 | 0.0030 | -0.80 |
| Age:Education Missing pre 1920 | 0.0048 | 0.0022 | 2.17 |
| Education Short:Calendar time | 0.0005 | 0.0018 | 0.28 |
| Education Medium:Calendar time | -0.0057 | 0.0032 | -1.75 |
| Education Long:Calendar time | 0.0029 | 0.0038 | 0.76 |
| Education Missing:Calendar time | 0.0088 | 0.0057 | 1.54 |
| Education Missing pre 1920:Calendar time | -0.0094 | 0.0026 | -3.67 |
| Calendar time:Occupation Employed | 0.0144 | 0.0026 | 5.50 |
| Calendar time:Occupation Early retirement pension | 0.0148 | 0.0026 | 5.77 |
| Calendar time:Occupation Missing | -0.0047 | 0.0925 | -0.05 |
| Calendar time:Occupation Other | 0.0108 | 0.0079 | 1.38 |
| Calendar time:Occupation Sick leave, etc. | 0.0046 | 0.0066 | 0.70 |
| Calendar time:Occupation Student | -0.0086 | 0.0311 | -0.27 |
| Calendar time:Occupation Unemployed | -0.0025 | 0.0095 | -0.27 |
| JointDisease:Dementia | -0.2829 | 0.0933 | -3.03 |
| Osteoarthritis:Dementia | -0.2786 | 0.0487 | -5.72 |
| Back pain:Dementia | -0.2097 | 0.0534 | -3.93 |
| Dementia:Schizophrenia | 0.3342 | 0.0496 | 6.74 |
| Schizophrenia:Depression | -0.4133 | 0.0443 | -9.33 |
| Cancer:Depression | -0.0714 | 0.0270 | -2.65 |
| COPD:Depression | -0.0883 | 0.0206 | -4.29 |
| COPD:Diabetes | -0.1981 | 0.0229 | -8.66 |
| Hypertension:High cholesterol | 0.0582 | 0.0209 | 2.79 |
| High cholesterol:Allergies | -0.0807 | 0.0160 | -5.04 |
| High cholesterol:Diabetes | 0.1478 | 0.0196 | 7.56 |
| Stroke:Diabetes | 0.0942 | 0.0235 | 4.00 |
| Sex Female:Stroke | -0.1880 | 0.0192 | -9.80 |
| Calendar time:Hypertension | -0.0271 | 0.0018 | -14.71 |
| Sex Female:High cholesterol | 0.1703 | 0.0156 | 10.89 |
| Age:High cholesterol | -0.0051 | 0.0008 | -6.45 |
| Sex Female:Back pain | -0.1224 | 0.0235 | -5.22 |
| Sex Female:Cancer | -0.1677 | 0.0227 | -7.40 |
| Sex Female:COPD | -0.2053 | 0.0177 | -11.60 |
| Age:COPD | -0.0088 | 0.0009 | -10.37 |
| Sex Female:Depression | -0.1754 | 0.0194 | -9.05 |
| Sex Female:Diabetes | -0.1254 | 0.0198 | -6.32 |
