# Supplementary material for: Longitudinal models for the progression of disease portfolios in a nationwide chronic heart disease population
Source: PLoS One. 2023 Apr 20;18(4):e0284496. doi: 10.1371/journal.pone.0284496 (PMC10118194; doi:10.1371/journal.pone.0284496)
Supplement: S9 Table — (DOCX) [file pone.0284496.s014.docx]

**Table S9: Parameter estimates for effects on obtaining osteoarthritis as the next chronic disease diagnosis.**

|  | Estimate | Std. Error | z value |
| --- | --- | --- | --- |
| (Intercept) | -3.3141 | 0.0225 | -147.24 |
| Sex Female | 0.1767 | 0.0234 | 7.56 |
| Age | -0.0289 | 0.0011 | -27.05 |
| Education Short | 0.0343 | 0.0187 | 1.83 |
| Education Medium | 0.0681 | 0.0338 | 2.02 |
| Education Long | 0.0307 | 0.0399 | 0.77 |
| Education Missing | -0.1455 | 0.0585 | -2.49 |
| Education Missing pre 1920 | 0.0130 | 0.0610 | 0.21 |
| Calendar time | 0.0242 | 0.0019 | 12.78 |
| Occupation Employed | 0.1417 | 0.0221 | 6.41 |
| Occupation Early retirement pension | -0.2093 | 0.0341 | -6.14 |
| Occupation Missing | -1.2142 | 0.7685 | -1.58 |
| Occupation Other | -0.1349 | 0.0780 | -1.73 |
| Occupation Sick leave, etc. | -0.0689 | 0.0716 | -0.96 |
| Occupation Student | 0.1896 | 0.3244 | 0.58 |
| Occupation Unemployed | 0.0647 | 0.1094 | 0.59 |
| Age^2 | -0.0006 | 0.0001 | -12.24 |
| Calendar time^2 | -0.0029 | 0.0002 | -17.46 |
| Stroke | -0.3110 | 0.0220 | -14.12 |
| Hypertension | 0.3750 | 0.0194 | 19.31 |
| High cholesterol | 0.5358 | 0.0258 | 20.73 |
| Allergies | 0.2878 | 0.0233 | 12.33 |
| JointDisease | 0.4391 | 0.0225 | 19.56 |
| Osteoporosis | 0.2354 | 0.0143 | 16.49 |
| Back pain | 0.5132 | 0.0143 | 35.99 |
| COPD | -0.1407 | 0.0137 | -10.29 |
| Dementia | -0.5671 | 0.0490 | -11.56 |
| Schizophrenia | -0.2795 | 0.0374 | -7.47 |
| Depression | -0.0242 | 0.0189 | -1.28 |
| Diabetes | -0.3077 | 0.0216 | -14.24 |
| Sex Female:Calendar time | 0.0054 | 0.0017 | 3.10 |
| Age:Occupation Employed | 0.0141 | 0.0020 | 7.16 |
| Age:Occupation Early retirement pension | 0.0073 | 0.0027 | 2.73 |
| Age:Occupation Missing | -0.0393 | 0.0347 | -1.13 |
| Age:Occupation Other | 0.0075 | 0.0052 | 1.45 |
| Age:Occupation Sick leave, etc. | 0.0154 | 0.0039 | 3.93 |
| Age:Occupation Student | 0.0210 | 0.0125 | 1.67 |
| Age:Occupation Unemployed | 0.0176 | 0.0062 | 2.84 |
| Age:Education Short | -0.0012 | 0.0011 | -1.09 |
| Age:Education Medium | -0.0013 | 0.0018 | -0.76 |
| Age:Education Long | 0.0006 | 0.0022 | 0.29 |
| Age:Education Missing | -0.0001 | 0.0030 | -0.03 |
| Age:Education Missing pre 1920 | 0.0135 | 0.0037 | 3.70 |
| Education Short:Calendar time | 0.0063 | 0.0021 | 3.07 |
| Education Medium:Calendar time | 0.0065 | 0.0035 | 1.86 |
| Education Long:Calendar time | 0.0113 | 0.0042 | 2.69 |
| Education Missing:Calendar time | 0.0040 | 0.0061 | 0.65 |
| Education Missing pre 1920:Calendar time | -0.0052 | 0.0047 | -1.10 |
| Calendar time:Occupation Employed | -0.0097 | 0.0023 | -4.15 |
| Calendar time:Occupation Early retirement pension | -0.0100 | 0.0028 | -3.52 |
| Calendar time:Occupation Missing | -0.1312 | 0.0955 | -1.37 |
| Calendar time:Occupation Other | -0.0156 | 0.0076 | -2.06 |
| Calendar time:Occupation Sick leave, etc. | -0.0101 | 0.0057 | -1.79 |
| Calendar time:Occupation Student | -0.0231 | 0.0268 | -0.86 |
| Calendar time:Occupation Unemployed | -0.0065 | 0.0078 | -0.84 |
| COPD:Schizophrenia | 0.2513 | 0.0732 | 3.44 |
| Osteoporosis:Back pain | -0.1229 | 0.0318 | -3.86 |
| COPD:Diabetes | 0.1140 | 0.0293 | 3.89 |
| Hypertension:High cholesterol | 0.0789 | 0.0228 | 3.45 |
| High cholesterol:Allergies | -0.0934 | 0.0201 | -4.66 |
| High cholesterol:Diabetes | 0.3825 | 0.0254 | 15.08 |
| High cholesterol:Dementia | -0.2905 | 0.0716 | -4.06 |
| Stroke:Dementia | 0.3734 | 0.0766 | 4.87 |
| Stroke:High cholesterol | 0.0744 | 0.0276 | 2.70 |
| Sex Female:Hypertension | 0.1605 | 0.0230 | 6.99 |
| Sex Female:High cholesterol | -0.1623 | 0.0189 | -8.57 |
| Sex Female:Allergies | 0.0681 | 0.0198 | 3.44 |
| Sex Female:Depression | 0.1089 | 0.0239 | 4.56 |
| Education Short:High cholesterol | 0.0027 | 0.0212 | 0.13 |
| Education Medium:High cholesterol | 0.0211 | 0.0369 | 0.57 |
| Education Long:High cholesterol | 0.1184 | 0.0444 | 2.66 |
| Education Missing:High cholesterol | 0.1020 | 0.0638 | 1.60 |
| Education Missing pre 1920:High cholesterol | -0.1072 | 0.0482 | -2.22 |
| Education Short:Allergies | 0.0879 | 0.0220 | 3.99 |
| Education Medium:Allergies | 0.0629 | 0.0382 | 1.65 |
| Education Long:Allergies | 0.0820 | 0.0453 | 1.81 |
| Education Missing:Allergies | 0.2107 | 0.0645 | 3.27 |
| Education Missing pre 1920:Allergies | -0.0722 | 0.0343 | -2.11 |
