# Supplementary material for: Longitudinal models for the progression of disease portfolios in a nationwide chronic heart disease population
Source: PLoS One. 2023 Apr 20;18(4):e0284496. doi: 10.1371/journal.pone.0284496 (PMC10118194; doi:10.1371/journal.pone.0284496)
Supplement: S10 Table — (DOCX) [file pone.0284496.s015.docx]

Table S10: Parameter estimates for effects on obtaining back pain as the next chronic disease diagnosis.

|  | Estimate | Std. Error | z value |
| --- | --- | --- | --- |
| (Intercept) | -3.7207 | 0.0224 | -166.06 |
| Sex Female | 0.0799 | 0.0230 | 3.47 |
| Age | -0.0248 | 0.0011 | -22.46 |
| Education Short | 0.0848 | 0.0181 | 4.69 |
| Education Medium | 0.1640 | 0.0327 | 5.02 |
| Education Long | 0.2633 | 0.0367 | 7.17 |
| Education Missing | 0.1496 | 0.0529 | 2.83 |
| Education Missing pre 1920 | 0.2037 | 0.0406 | 5.02 |
| Calendar time | 0.0254 | 0.0023 | 10.95 |
| Occupation Employed | -0.0741 | 0.0259 | -2.86 |
| Occupation Early retirement pension | 0.1070 | 0.0343 | 3.12 |
| Occupation Missing | -0.5245 | 0.6812 | -0.77 |
| Occupation Other | -0.0506 | 0.0789 | -0.64 |
| Occupation Sick leave, etc. | 0.0740 | 0.0682 | 1.08 |
| Occupation Student | 0.2693 | 0.2418 | 1.11 |
| Occupation Unemployed | -0.2254 | 0.1091 | -2.07 |
| Age^2 | 0.0002 | 0.0000 | 3.16 |
| Calendar time^2 | -0.0006 | 0.0002 | -3.25 |
| Calendar time^3 | -0.0002 | 0.0000 | -5.73 |
| Stroke | -0.1234 | 0.0218 | -5.65 |
| Hypertension | 0.1863 | 0.0195 | 9.54 |
| High cholesterol | 0.3843 | 0.0262 | 14.66 |
| Allergies | 0.2802 | 0.0149 | 18.78 |
| JointDisease | 0.0978 | 0.0249 | 3.93 |
| Osteoporosis | 0.4532 | 0.0166 | 27.38 |
| Osteoarthritis | 0.6327 | 0.0143 | 44.37 |
| Cancer | 0.3557 | 0.0210 | 16.93 |
| COPD | 0.2060 | 0.0135 | 15.26 |
| Dementia | -0.4609 | 0.0409 | -11.27 |
| Schizophrenia | -0.1832 | 0.0325 | -5.64 |
| Depression | 0.2505 | 0.0120 | 20.90 |
| Diabetes | -0.2214 | 0.0216 | -10.26 |
| Age:Occupation Employed | -0.0099 | 0.0021 | -4.63 |
| Age:Occupation Early retirement pension | 0.0072 | 0.0026 | 2.78 |
| Age:Occupation Missing | -0.0068 | 0.0298 | -0.23 |
| Age:Occupation Other | -0.0041 | 0.0047 | -0.87 |
| Age:Occupation Sick leave, etc. | -0.0024 | 0.0036 | -0.67 |
| Age:Occupation Student | 0.0076 | 0.0073 | 1.04 |
| Age:Occupation Unemployed | -0.0130 | 0.0055 | -2.37 |
| Age:Education Short | 0.0042 | 0.0010 | 4.17 |
| Age:Education Medium | 0.0043 | 0.0017 | 2.58 |
| Age:Education Long | 0.0110 | 0.0021 | 5.14 |
| Age:Education Missing | 0.0019 | 0.0026 | 0.74 |
| Age:Education Missing pre 1920 | 0.0001 | 0.0032 | 0.02 |
| Calendar time:Occupation Employed | 0.0181 | 0.0024 | 7.57 |
| Calendar time:Occupation Early retirement pension | -0.0023 | 0.0029 | -0.79 |
| Calendar time:Occupation Missing | -0.0196 | 0.0923 | -0.21 |
| Calendar time:Occupation Other | 0.0139 | 0.0077 | 1.81 |
| Calendar time:Occupation Sick leave, etc. | 0.0225 | 0.0052 | 4.36 |
| Calendar time:Occupation Student | 0.0125 | 0.0207 | 0.60 |
| Calendar time:Occupation Unemployed | 0.0230 | 0.0078 | 2.93 |
| Osteoporosis:Osteoarthritis | -0.1756 | 0.0313 | -5.62 |
| Osteoporosis:COPD | 0.1173 | 0.0286 | 4.10 |
| Hypertension:High cholesterol | 0.2197 | 0.0236 | 9.30 |
| High cholesterol:Diabetes | 0.2929 | 0.0263 | 11.12 |
| Stroke:Dementia | 0.2223 | 0.0731 | 3.04 |
| Stroke:High cholesterol | 0.0715 | 0.0277 | 2.59 |
| Sex Female:Hypertension | 0.1074 | 0.0236 | 4.55 |
| Sex Female:High cholesterol | -0.1524 | 0.0194 | -7.86 |
| Education Short:High cholesterol | -0.0197 | 0.0221 | -0.89 |
| Education Medium:High cholesterol | -0.0610 | 0.0386 | -1.58 |
| Education Long:High cholesterol | -0.1582 | 0.0465 | -3.40 |
| Education Missing:High cholesterol | -0.1198 | 0.0628 | -1.91 |
| Education Missing pre 1920:High cholesterol | -0.1851 | 0.0490 | -3.78 |
| Sex Female:Allergies | 0.0882 | 0.0201 | 4.38 |
| Sex Female:Cancer | -0.1706 | 0.0300 | -5.68 |
