# Supplementary material for: Longitudinal models for the progression of disease portfolios in a nationwide chronic heart disease population
Source: PLoS One. 2023 Apr 20;18(4):e0284496. doi: 10.1371/journal.pone.0284496 (PMC10118194; doi:10.1371/journal.pone.0284496)
Supplement: S11 Table — (DOCX) [file pone.0284496.s016.docx]

**Table S11: Parameter estimates for effects on obtaining cancer as the next chronic disease diagnosis.**

|  | Estimate | Std. Error | z value |
| --- | --- | --- | --- |
| (Intercept) | -2.2875 | 0.0124 | -185.12 |
| Sex Female | -0.3832 | 0.0089 | -43.12 |
| Age | 0.0072 | 0.0009 | 8.20 |
| Education Short | 0.0685 | 0.0110 | 6.22 |
| Education Medium | 0.1389 | 0.0196 | 7.07 |
| Education Long | 0.1069 | 0.0220 | 4.86 |
| Education Missing | -0.1888 | 0.0382 | -4.95 |
| Education Missing pre 1920 | 0.1360 | 0.0258 | 5.27 |
| Calendar time | -0.0004 | 0.0011 | -0.35 |
| Occupation Employed | -0.0207 | 0.0172 | -1.21 |
| Occupation Early retirement pension | -0.0823 | 0.0301 | -2.74 |
| Occupation Missing | -1.4467 | 0.8185 | -1.77 |
| Occupation Other | -0.0145 | 0.0662 | -0.22 |
| Occupation Sick leave, etc. | -0.1218 | 0.0655 | -1.86 |
| Occupation Student | -0.5679 | 0.3155 | -1.80 |
| Occupation Unemployed | 0.0212 | 0.1085 | 0.20 |
| Age^2 | -0.0005 | 0.0000 | -11.28 |
| Stroke | -0.1902 | 0.0150 | -12.68 |
| Hypertension | 0.3132 | 0.0089 | 35.28 |
| High cholesterol | 0.2902 | 0.0092 | 31.57 |
| Allergies | 0.0217 | 0.0083 | 2.63 |
| JointDisease | -0.0991 | 0.0207 | -4.79 |
| Osteoporosis | 0.0457 | 0.0137 | 3.33 |
| Osteoarthritis | 0.0305 | 0.0113 | 2.71 |
| Back pain | -0.0590 | 0.0139 | -4.24 |
| COPD | 0.2638 | 0.0146 | 18.08 |
| Dementia | -0.6258 | 0.0621 | -10.08 |
| Schizophrenia | -0.3402 | 0.0346 | -9.83 |
| Depression | -0.1347 | 0.0161 | -8.38 |
| Diabetes | -0.1006 | 0.0187 | -5.38 |
| Sex Female:Calendar time | 0.0070 | 0.0014 | 5.15 |
| Age:Occupation Employed | 0.0178 | 0.0016 | 11.38 |
| Age:Occupation Early retirement pension | 0.0121 | 0.0025 | 4.91 |
| Age:Occupation Missing | 0.0465 | 0.0732 | 0.63 |
| Age:Occupation Other | 0.0190 | 0.0051 | 3.73 |
| Age:Occupation Sick leave, etc. | 0.0201 | 0.0039 | 5.21 |
| Age:Occupation Student | -0.0222 | 0.0114 | -1.95 |
| Age:Occupation Unemployed | 0.0228 | 0.0067 | 3.42 |
| Age:Education Short | 0.0025 | 0.0009 | 2.65 |
| Age:Education Medium | 0.0028 | 0.0016 | 1.74 |
| Age:Education Long | 0.0008 | 0.0019 | 0.40 |
| Age:Education Missing | 0.0016 | 0.0029 | 0.55 |
| Age:Education Missing pre 1920 | -0.0063 | 0.0022 | -2.83 |
| Calendar time:Occupation Employed | -0.0080 | 0.0021 | -3.89 |
| Calendar time:Occupation Early retirement pension | -0.0065 | 0.0025 | -2.57 |
| Calendar time:Occupation Missing | 0.0596 | 0.1368 | 0.44 |
| Calendar time:Occupation Other | -0.0253 | 0.0069 | -3.65 |
| Calendar time:Occupation Sick leave, etc. | -0.0229 | 0.0058 | -3.96 |
| Calendar time:Occupation Student | -0.0152 | 0.0269 | -0.57 |
| Calendar time:Occupation Unemployed | -0.0194 | 0.0077 | -2.52 |
| Osteoporosis:COPD | 0.1111 | 0.0237 | 4.69 |
| COPD:Schizophrenia | 0.2116 | 0.0596 | 3.55 |
| Dementia:Schizophrenia | 0.3607 | 0.0731 | 4.94 |
| COPD:Depression | 0.1146 | 0.0226 | 5.07 |
| Osteoporosis:Back pain | 0.1142 | 0.0292 | 3.92 |
| High cholesterol:Diabetes | 0.2688 | 0.0201 | 13.35 |
| Stroke:Dementia | 0.3155 | 0.0481 | 6.56 |
| Hypertension:Dementia | 0.2394 | 0.0647 | 3.70 |
| Stroke:High cholesterol | 0.1229 | 0.0202 | 6.09 |
| Sex Female:Depression | 0.1074 | 0.0200 | 5.36 |
| Age:Osteoporosis | -0.0073 | 0.0011 | -6.72 |
| Education Short:Diabetes | -0.0153 | 0.0215 | -0.71 |
| Education Medium:Diabetes | -0.1263 | 0.0417 | -3.03 |
| Education Long:Diabetes | 0.0472 | 0.0493 | 0.96 |
| Education Missing:Diabetes | -0.0819 | 0.0621 | -1.32 |
| Education Missing pre 1920:Diabetes | 0.1307 | 0.0301 | 4.35 |
| Education Short:COPD | -0.0272 | 0.0205 | -1.33 |
| Education Medium:COPD | -0.0772 | 0.0410 | -1.88 |
| Education Long:COPD | -0.1371 | 0.0528 | -2.60 |
| Education Missing:COPD | 0.1737 | 0.0649 | 2.67 |
| Education Missing pre 1920:COPD | -0.0766 | 0.0289 | -2.65 |
