# Supplementary material for: Longitudinal models for the progression of disease portfolios in a nationwide chronic heart disease population
Source: PLoS One. 2023 Apr 20;18(4):e0284496. doi: 10.1371/journal.pone.0284496 (PMC10118194; doi:10.1371/journal.pone.0284496)
Supplement: S12 Table — (DOCX) [file pone.0284496.s017.docx]

**Table S12: Parameter estimates for effects on obtaining COPD as the next chronic disease diagnosis.**

|  | Estimate | Std. Error | z value |
| --- | --- | --- | --- |
| (Intercept) | -2.1874 | 0.0163 | -134.61 |
| Sex Female | -0.2859 | 0.0175 | -16.32 |
| Age | 0.0012 | 0.0009 | 1.29 |
| Education Short | -0.1887 | 0.0135 | -13.98 |
| Education Medium | -0.3701 | 0.0265 | -13.97 |
| Education Long | -0.5687 | 0.0322 | -17.65 |
| Education Missing | -0.0978 | 0.0400 | -2.44 |
| Education Missing pre 1920 | 0.4925 | 0.0416 | 11.85 |
| Calendar time | 0.0211 | 0.0019 | 11.13 |
| Occupation Employed | -0.2542 | 0.0182 | -13.94 |
| Occupation Early retirement pension | 0.1825 | 0.0246 | 7.42 |
| Occupation Missing | 0.0636 | 0.3818 | 0.17 |
| Occupation Other | -0.1217 | 0.0625 | -1.95 |
| Occupation Sick leave, etc. | -0.0864 | 0.0561 | -1.54 |
| Occupation Student | 0.6720 | 0.2848 | 2.36 |
| Occupation Unemployed | -0.1811 | 0.0914 | -1.98 |
| Age^2 | -0.0002 | 0.0000 | -3.75 |
| Calendar time^2 | 0.0023 | 0.0001 | 18.23 |
| Stroke | -0.2155 | 0.0151 | -14.32 |
| Hypertension | 0.3866 | 0.0145 | 26.75 |
| High cholesterol | -0.0153 | 0.0207 | -0.74 |
| Allergies | 0.2207 | 0.0120 | 18.45 |
| JointDisease | 0.0319 | 0.0225 | 1.42 |
| Osteoporosis | 0.3657 | 0.0111 | 32.97 |
| Osteoarthritis | -0.0783 | 0.0127 | -6.14 |
| Back pain | -0.0001 | 0.0142 | -0.01 |
| Cancer | 0.3731 | 0.0148 | 25.17 |
| Dementia | -0.3469 | 0.0308 | -11.25 |
| Schizophrenia | 0.2466 | 0.0282 | 8.74 |
| Depression | 0.2378 | 0.0094 | 25.25 |
| Diabetes | -0.2531 | 0.0148 | -17.10 |
| Sex Female:Calendar time | 0.0125 | 0.0013 | 9.48 |
| Age:Occupation Employed | 0.0088 | 0.0016 | 5.55 |
| Age:Occupation Early retirement pension | 0.0075 | 0.0021 | 3.62 |
| Age:Occupation Missing | 0.0290 | 0.0285 | 1.02 |
| Age:Occupation Other | 0.0081 | 0.0043 | 1.90 |
| Age:Occupation Sick leave, etc. | 0.0094 | 0.0031 | 3.02 |
| Age:Occupation Student | 0.0432 | 0.0119 | 3.64 |
| Age:Occupation Unemployed | 0.0052 | 0.0052 | 1.00 |
| Age:Education Short | 0.0030 | 0.0008 | 3.61 |
| Age:Education Medium | 0.0020 | 0.0015 | 1.31 |
| Age:Education Long | 0.0000 | 0.0020 | 0.02 |
| Age:Education Missing | 0.0046 | 0.0023 | 1.96 |
| Age:Education Missing pre 1920 | -0.0456 | 0.0026 | -17.58 |
| Education Short:Calendar time | 0.0042 | 0.0015 | 2.74 |
| Education Medium:Calendar time | 0.0023 | 0.0028 | 0.82 |
| Education Long:Calendar time | 0.0106 | 0.0035 | 3.00 |
| Education Missing:Calendar time | 0.0044 | 0.0045 | 0.98 |
| Education Missing pre 1920:Calendar time | 0.0464 | 0.0032 | 14.41 |
| Osteoarthritis:Dementia | 0.3021 | 0.0563 | 5.36 |
| Dementia:Schizophrenia | 0.1791 | 0.0606 | 2.96 |
| Schizophrenia:Depression | -0.1737 | 0.0442 | -3.93 |
| Osteoporosis:Back pain | 0.0845 | 0.0268 | 3.15 |
| Osteoarthritis:Back pain | 0.2036 | 0.0272 | 7.49 |
| JointDisease:Osteoarthritis | 0.1494 | 0.0434 | 3.44 |
| Hypertension:High cholesterol | 0.0782 | 0.0188 | 4.16 |
| High cholesterol:Diabetes | 0.3967 | 0.0188 | 21.13 |
| High cholesterol:Dementia | -0.1176 | 0.0437 | -2.69 |
| Stroke:Dementia | 0.5001 | 0.0440 | 11.36 |
| Stroke:Diabetes | 0.0783 | 0.0235 | 3.33 |
| Stroke:High cholesterol | 0.2536 | 0.0199 | 12.77 |
| Sex Female:Hypertension | -0.0723 | 0.0178 | -4.06 |
| Sex Female:High cholesterol | 0.0761 | 0.0150 | 5.07 |
| Sex Female:Cancer | -0.1055 | 0.0223 | -4.74 |
| Age:High cholesterol | -0.0127 | 0.0008 | -16.47 |
| Age:Osteoporosis | -0.0064 | 0.0010 | -6.79 |
| Age:Osteoarthritis | -0.0043 | 0.0010 | -4.16 |
| Age:Cancer | -0.0052 | 0.0011 | -4.67 |
| Education Short:High cholesterol | 0.0843 | 0.0171 | 4.92 |
| Education Medium:High cholesterol | 0.1294 | 0.0321 | 4.03 |
| Education Long:High cholesterol | 0.0615 | 0.0403 | 1.53 |
| Education Missing:High cholesterol | -0.0114 | 0.0494 | -0.23 |
| Education Missing pre 1920:High cholesterol | -0.1470 | 0.0396 | -3.71 |
| Education Short:Allergies | 0.0300 | 0.0179 | 1.67 |
| Education Medium:Allergies | 0.0754 | 0.0336 | 2.24 |
| Education Long:Allergies | 0.1373 | 0.0413 | 3.32 |
| Education Missing:Allergies | 0.0923 | 0.0523 | 1.77 |
| Education Missing pre 1920:Allergies | -0.0103 | 0.0238 | -0.43 |
| Calendar time:Hypertension | -0.0177 | 0.0016 | -10.94 |
| Calendar time:High cholesterol | 0.0095 | 0.0014 | 6.69 |
| Calendar time:Osteoarthritis | 0.0072 | 0.0019 | 3.74 |
| Calendar time:Cancer | 0.0070 | 0.0019 | 3.62 |
