# Supplementary material for: Longitudinal models for the progression of disease portfolios in a nationwide chronic heart disease population
Source: PLoS One. 2023 Apr 20;18(4):e0284496. doi: 10.1371/journal.pone.0284496 (PMC10118194; doi:10.1371/journal.pone.0284496)
Supplement: S13 Table — (DOCX) [file pone.0284496.s018.docx]

**Table S13: Parameter estimates for effects on obtaining schizophrenia as the next chronic disease diagnosis.**

|  | Estimate | Std. Error | z value |
| --- | --- | --- | --- |
| (Intercept) | -5.0016 | 0.0316 | -158.26 |
| Sex Female | -0.1561 | 0.0175 | -8.92 |
| Age | 0.0368 | 0.0019 | 19.42 |
| Education Short | -0.0997 | 0.0214 | -4.66 |
| Education Medium | -0.1753 | 0.0422 | -4.15 |
| Education Long | -0.0471 | 0.0459 | -1.03 |
| Education Missing | 0.1737 | 0.0620 | 2.80 |
| Education Missing pre 1920 | 0.3304 | 0.0595 | 5.55 |
| Calendar time | -0.0549 | 0.0046 | -11.88 |
| Occupation Employed | -0.4051 | 0.0494 | -8.20 |
| Occupation Early retirement pension | 0.3911 | 0.0584 | 6.69 |
| Occupation Missing | 1.5373 | 0.5231 | 2.94 |
| Occupation Other | -0.2657 | 0.1546 | -1.72 |
| Occupation Sick leave, etc. | -0.0802 | 0.1215 | -0.66 |
| Occupation Student | -0.5856 | 0.6051 | -0.97 |
| Occupation Unemployed | -1.0214 | 0.2677 | -3.82 |
| Age^2 | 0.0011 | 0.0001 | 14.14 |
| Calendar time^2 | -0.0016 | 0.0003 | -5.52 |
| Calendar time^3 | 0.0005 | 0.0001 | 9.01 |
| Stroke | 0.4011 | 0.0465 | 8.63 |
| Hypertension | 0.2785 | 0.0242 | 11.49 |
| High cholesterol | -0.1470 | 0.0198 | -7.41 |
| Allergies | 0.0968 | 0.0164 | 5.89 |
| JointDisease | -0.1551 | 0.0442 | -3.51 |
| Osteoporosis | 0.4625 | 0.0183 | 25.34 |
| Osteoarthritis | -0.0524 | 0.0268 | -1.95 |
| Back pain | -0.1844 | 0.0626 | -2.95 |
| Cancer | 0.3109 | 0.0301 | 10.31 |
| COPD | 0.0915 | 0.0196 | 4.66 |
| Dementia | 2.5673 | 0.0243 | 105.45 |
| Depression | 1.2490 | 0.0375 | 33.29 |
| Diabetes | 0.0542 | 0.0231 | 2.34 |
| Sex Female:Calendar time | -0.0233 | 0.0029 | -7.93 |
| Age:Occupation Employed | -0.0162 | 0.0036 | -4.43 |
| Age:Occupation Early retirement pension | -0.0201 | 0.0045 | -4.43 |
| Age:Occupation Missing | 0.0027 | 0.0250 | 0.11 |
| Age:Occupation Other | -0.0396 | 0.0079 | -5.00 |
| Age:Occupation Sick leave, etc. | -0.0432 | 0.0060 | -7.16 |
| Age:Occupation Student | -0.0216 | 0.0138 | -1.57 |
| Age:Occupation Unemployed | -0.0597 | 0.0110 | -5.44 |
| Age:Education Short | -0.0045 | 0.0017 | -2.62 |
| Age:Education Medium | -0.0130 | 0.0030 | -4.27 |
| Age:Education Long | -0.0113 | 0.0038 | -2.93 |
| Age:Education Missing | 0.0037 | 0.0041 | 0.91 |
| Age:Education Missing pre 1920 | -0.0213 | 0.0041 | -5.19 |
| Education Short:Calendar time | 0.0097 | 0.0038 | 2.53 |
| Education Medium:Calendar time | 0.0195 | 0.0071 | 2.74 |
| Education Long:Calendar time | -0.0004 | 0.0083 | -0.05 |
| Education Missing:Calendar time | -0.0006 | 0.0103 | -0.06 |
| Education Missing pre 1920:Calendar time | 0.0159 | 0.0050 | 3.18 |
| Calendar time:Occupation Employed | 0.0436 | 0.0062 | 6.98 |
| Calendar time:Occupation Early retirement pension | 0.0354 | 0.0051 | 7.00 |
| Calendar time:Occupation Missing | -0.0007 | 0.0882 | -0.01 |
| Calendar time:Occupation Other | 0.0429 | 0.0160 | 2.68 |
| Calendar time:Occupation Sick leave, etc. | 0.0792 | 0.0089 | 8.87 |
| Calendar time:Occupation Student | 0.1825 | 0.0373 | 4.89 |
| Calendar time:Occupation Unemployed | 0.0750 | 0.0189 | 3.97 |
| Osteoarthritis:Dementia | -0.1956 | 0.0552 | -3.55 |
| Back pain:Dementia | -0.2812 | 0.0595 | -4.72 |
| Cancer:Depression | -0.2068 | 0.0492 | -4.20 |
| Back pain:Cancer | 0.2469 | 0.0660 | 3.74 |
| High cholesterol:Dementia | 0.2172 | 0.0393 | 5.53 |
| Stroke:Dementia | -0.3968 | 0.0400 | -9.92 |
| Stroke:Diabetes | 0.1186 | 0.0421 | 2.82 |
| Hypertension:Back pain | 0.1787 | 0.0663 | 2.69 |
| Hypertension:Depression | -0.2009 | 0.0407 | -4.94 |
| Stroke:Hypertension | 0.1333 | 0.0488 | 2.73 |
