# Supplementary material for: Longitudinal models for the progression of disease portfolios in a nationwide chronic heart disease population
Source: PLoS One. 2023 Apr 20;18(4):e0284496. doi: 10.1371/journal.pone.0284496 (PMC10118194; doi:10.1371/journal.pone.0284496)
Supplement: S14 Table — (DOCX) [file pone.0284496.s019.docx]

Table S14: Parameter estimates for effects on obtaining dementia as the next chronic disease diagnosis.

|  | Estimate | Std. Error | z value |
| --- | --- | --- | --- |
| (Intercept) | -3.8808 | 0.0232 | -167.22 |
| Sex Female | -0.1118 | 0.0106 | -10.56 |
| Age | 0.0989 | 0.0016 | 61.27 |
| Education Short | -0.0089 | 0.0155 | -0.58 |
| Education Medium | -0.0170 | 0.0292 | -0.58 |
| Education Long | 0.0815 | 0.0327 | 2.49 |
| Education Missing | -0.0112 | 0.0467 | -0.24 |
| Education Missing pre 1920 | 0.7835 | 0.0412 | 19.02 |
| Calendar time | -0.0323 | 0.0031 | -10.37 |
| Occupation Employed | -0.4210 | 0.0328 | -12.82 |
| Occupation Early retirement pension | -0.2029 | 0.0720 | -2.82 |
| Occupation Missing | -3.5679 | 3.9496 | -0.90 |
| Occupation Other | -0.1988 | 0.1208 | -1.65 |
| Occupation Sick leave, etc. | -0.3273 | 0.1142 | -2.87 |
| Occupation Student | -11.7761 | 55.3133 | -0.21 |
| Occupation Unemployed | -1.0799 | 0.5341 | -2.02 |
| Age^2 | -0.0004 | 0.0001 | -4.65 |
| Calendar time^2 | -0.0013 | 0.0002 | -6.44 |
| Stroke | 0.7035 | 0.0137 | 51.37 |
| Hypertension | 0.2215 | 0.0193 | 11.45 |
| High cholesterol | 0.2070 | 0.0307 | 6.74 |
| Allergies | 0.1023 | 0.0140 | 7.30 |
| JointDisease | -0.1393 | 0.0288 | -4.83 |
| Osteoporosis | 0.5971 | 0.0130 | 46.08 |
| Osteoarthritis | 0.0835 | 0.0188 | 4.43 |
| Back pain | -0.0237 | 0.0198 | -1.20 |
| COPD | -0.0177 | 0.0140 | -1.27 |
| Schizophrenia | 2.1602 | 0.0272 | 79.48 |
| Depression | 0.7324 | 0.0122 | 60.12 |
| Diabetes | 0.1281 | 0.0176 | 7.28 |
| Age:Occupation Employed | 0.0334 | 0.0030 | 11.31 |
| Age:Occupation Early retirement pension | 0.0234 | 0.0064 | 3.63 |
| Age:Occupation Missing | 0.2512 | 0.2823 | 0.89 |
| Age:Occupation Other | 0.0130 | 0.0120 | 1.08 |
| Age:Occupation Sick leave, etc. | 0.0547 | 0.0095 | 5.76 |
| Age:Occupation Student | -0.1401 | 1.5655 | -0.09 |
| Age:Occupation Unemployed | 0.0038 | 0.0367 | 0.10 |
| Age:Education Short | 0.0028 | 0.0020 | 1.35 |
| Age:Education Medium | 0.0080 | 0.0039 | 2.07 |
| Age:Education Long | -0.0070 | 0.0039 | -1.79 |
| Age:Education Missing | 0.0077 | 0.0068 | 1.13 |
| Age:Education Missing pre 1920 | -0.0597 | 0.0032 | -18.57 |
| Education Short:Calendar time | -0.0020 | 0.0028 | -0.71 |
| Education Medium:Calendar time | -0.0139 | 0.0054 | -2.59 |
| Education Long:Calendar time | -0.0017 | 0.0057 | -0.29 |
| Education Missing:Calendar time | -0.0060 | 0.0093 | -0.65 |
| Education Missing pre 1920:Calendar time | 0.0630 | 0.0032 | 19.45 |
| Calendar time:Occupation Employed | -0.0253 | 0.0058 | -4.35 |
| Calendar time:Occupation Early retirement pension | -0.0091 | 0.0064 | -1.42 |
| Calendar time:Occupation Missing | -0.3125 | 0.3630 | -0.86 |
| Calendar time:Occupation Other | 0.0074 | 0.0187 | 0.40 |
| Calendar time:Occupation Sick leave, etc. | -0.0067 | 0.0200 | -0.33 |
| Calendar time:Occupation Student | 0.0342 | 4.8617 | 0.01 |
| Calendar time:Occupation Unemployed | -0.0356 | 0.0416 | -0.86 |
| Osteoporosis:Osteoarthritis | -0.1176 | 0.0318 | -3.69 |
| Back pain:Schizophrenia | -0.1714 | 0.0662 | -2.59 |
| COPD:Schizophrenia | -0.1551 | 0.0527 | -2.94 |
| Schizophrenia:Depression | -0.8147 | 0.0436 | -18.67 |
| Osteoarthritis:Back pain | 0.1007 | 0.0382 | 2.64 |
| Hypertension:High cholesterol | 0.0998 | 0.0320 | 3.12 |
| High cholesterol:Allergies | -0.0594 | 0.0228 | -2.60 |
| High cholesterol:Diabetes | 0.1553 | 0.0259 | 6.00 |
| Age:Stroke | -0.0209 | 0.0014 | -15.30 |
| Calendar time:Hypertension | -0.0237 | 0.0028 | -8.40 |
