# Supplementary material for: Longitudinal models for the progression of disease portfolios in a nationwide chronic heart disease population
Source: PLoS One. 2023 Apr 20;18(4):e0284496. doi: 10.1371/journal.pone.0284496 (PMC10118194; doi:10.1371/journal.pone.0284496)
Supplement: S15 Table — (DOCX) [file pone.0284496.s020.docx]

**Table S15: Parameter estimates for effects on obtaining depression as the next chronic disease diagnosis.**

|  | Estimate | Std. Error | z value |
| --- | --- | --- | --- |
| (Intercept) | -3.4370 | 0.0173 | -198.53 |
| Sex Female | 0.4799 | 0.0138 | 34.66 |
| Age | 0.0165 | 0.0010 | 16.79 |
| Education Short | -0.0668 | 0.0110 | -6.09 |
| Education Medium | -0.0820 | 0.0208 | -3.93 |
| Education Long | -0.0447 | 0.0240 | -1.87 |
| Education Missing | -0.1273 | 0.0341 | -3.73 |
| Education Missing pre 1920 | 0.4572 | 0.0365 | 12.51 |
| Calendar time | -0.0372 | 0.0019 | -19.38 |
| Occupation Employed | -0.1518 | 0.0225 | -6.74 |
| Occupation Early retirement pension | 0.2940 | 0.0284 | 10.35 |
| Occupation Missing | -0.4124 | 0.5643 | -0.73 |
| Occupation Other | -0.1351 | 0.0681 | -1.98 |
| Occupation Sick leave, etc. | 0.0613 | 0.0571 | 1.07 |
| Occupation Student | 0.9800 | 0.2027 | 4.84 |
| Occupation Unemployed | -0.2957 | 0.0918 | -3.22 |
| Age^2 | 0.0004 | 0.0000 | 9.92 |
| Calendar time^2 | -0.0012 | 0.0001 | -8.37 |
| Stroke | 0.7168 | 0.0254 | 28.22 |
| Hypertension | 0.3739 | 0.0126 | 29.78 |
| High cholesterol | 0.2961 | 0.0209 | 14.15 |
| Allergies | 0.4352 | 0.0152 | 28.59 |
| JointDisease | -0.1376 | 0.0219 | -6.28 |
| Osteoporosis | 0.4688 | 0.0127 | 36.87 |
| Osteoarthritis | 0.0414 | 0.0135 | 3.07 |
| Back pain | 0.5000 | 0.0269 | 18.62 |
| Cancer | 0.3558 | 0.0178 | 20.01 |
| COPD | 0.3284 | 0.0143 | 22.97 |
| Diabetes | 0.0638 | 0.0182 | 3.51 |
| Sex Female:Calendar time | -0.0058 | 0.0015 | -3.75 |
| Age:Occupation Employed | -0.0349 | 0.0019 | -18.71 |
| Age:Occupation Early retirement pension | -0.0192 | 0.0022 | -8.68 |
| Age:Occupation Missing | -0.0372 | 0.0252 | -1.48 |
| Age:Occupation Other | -0.0397 | 0.0040 | -9.89 |
| Age:Occupation Sick leave, etc. | -0.0433 | 0.0030 | -14.32 |
| Age:Occupation Student | 0.0060 | 0.0064 | 0.93 |
| Age:Occupation Unemployed | -0.0505 | 0.0046 | -11.08 |
| Age:Education Short | -0.0040 | 0.0008 | -4.68 |
| Age:Education Medium | -0.0085 | 0.0015 | -5.82 |
| Age:Education Long | -0.0085 | 0.0018 | -4.66 |
| Age:Education Missing | -0.0021 | 0.0023 | -0.92 |
| Age:Education Missing pre 1920 | -0.0316 | 0.0024 | -13.07 |
| Education Short:Calendar time | -0.0011 | 0.0018 | -0.60 |
| Education Medium:Calendar time | 0.0030 | 0.0032 | 0.93 |
| Education Long:Calendar time | -0.0116 | 0.0039 | -2.97 |
| Education Missing:Calendar time | 0.0129 | 0.0052 | 2.47 |
| Education Missing pre 1920:Calendar time | 0.0264 | 0.0031 | 8.46 |
| Calendar time:Occupation Employed | 0.0441 | 0.0022 | 19.70 |
| Calendar time:Occupation Early retirement pension | 0.0187 | 0.0026 | 7.34 |
| Calendar time:Occupation Missing | 0.0964 | 0.0694 | 1.39 |
| Calendar time:Occupation Other | 0.0397 | 0.0066 | 5.98 |
| Calendar time:Occupation Sick leave, etc. | 0.0645 | 0.0042 | 15.23 |
| Calendar time:Occupation Student | 0.0616 | 0.0179 | 3.43 |
| Calendar time:Occupation Unemployed | 0.0462 | 0.0066 | 6.98 |
| Osteoporosis:COPD | 0.1339 | 0.0242 | 5.54 |
| Osteoporosis:Back pain | -0.1020 | 0.0277 | -3.68 |
| Osteoarthritis:Back pain | 0.1157 | 0.0290 | 4.00 |
| Hypertension:High cholesterol | 0.0507 | 0.0197 | 2.58 |
| High cholesterol:Allergies | -0.0806 | 0.0168 | -4.79 |
| High cholesterol:Diabetes | 0.1652 | 0.0199 | 8.28 |
| Hypertension:Back pain | -0.1241 | 0.0291 | -4.27 |
| Stroke:Hypertension | 0.1657 | 0.0262 | 6.32 |
| Sex Female:Stroke | -0.2099 | 0.0186 | -11.29 |
| Sex Female:High cholesterol | -0.1336 | 0.0166 | -8.06 |
| Sex Female:Allergies | -0.0703 | 0.0168 | -4.18 |
| Sex Female:Cancer | -0.1213 | 0.0248 | -4.90 |
| Sex Female:COPD | -0.0865 | 0.0197 | -4.39 |
| Sex Female:Diabetes | -0.0938 | 0.0198 | -4.73 |
| Age:High cholesterol | -0.0080 | 0.0007 | -11.25 |
| Calendar time:High cholesterol | 0.0206 | 0.0016 | 12.69 |
