# Supplementary material for: Longitudinal models for the progression of disease portfolios in a nationwide chronic heart disease population
Source: PLoS One. 2023 Apr 20;18(4):e0284496. doi: 10.1371/journal.pone.0284496 (PMC10118194; doi:10.1371/journal.pone.0284496)
Supplement: S16 Table — (DOCX) [file pone.0284496.s021.docx]

Table S16: Parameter estimates for effects on obtaining diabetes as the next chronic disease diagnosis.

|  | Estimate | Std. Error | z value |
| --- | --- | --- | --- |
| (Intercept) | -3.1177 | 0.0188 | -165.90 |
| Sex Female | -0.4608 | 0.0222 | -20.75 |
| Age | -0.0076 | 0.0009 | -8.46 |
| Education Short | -0.1558 | 0.0105 | -14.90 |
| Education Medium | -0.2515 | 0.0189 | -13.29 |
| Education Long | -0.3204 | 0.0229 | -14.00 |
| Education Missing | 0.2432 | 0.0277 | 8.79 |
| Education Missing pre 1920 | -0.0892 | 0.0282 | -3.17 |
| Calendar time | 0.0034 | 0.0024 | 1.38 |
| Occupation Employed | -0.0180 | 0.0205 | -0.88 |
| Occupation Early retirement pension | 0.1199 | 0.0285 | 4.21 |
| Occupation Missing | 0.8375 | 0.3590 | 2.33 |
| Occupation Other | 0.1657 | 0.0660 | 2.51 |
| Occupation Sick leave, etc. | 0.2576 | 0.0579 | 4.45 |
| Occupation Student | -0.0083 | 0.3071 | -0.03 |
| Occupation Unemployed | 0.0266 | 0.0918 | 0.29 |
| Age^2 | -0.0004 | 0.0000 | -9.06 |
| Calendar time^2 | 0.0023 | 0.0002 | 14.64 |
| Calendar time^3 | -0.0003 | 0.0000 | -10.95 |
| Stroke | -0.2155 | 0.0156 | -13.78 |
| Hypertension | 0.7983 | 0.0167 | 47.93 |
| High cholesterol | 0.6729 | 0.0214 | 31.40 |
| Allergies | -0.0666 | 0.0133 | -5.01 |
| JointDisease | 0.1405 | 0.0224 | 6.27 |
| Osteoporosis | -0.3603 | 0.0179 | -20.08 |
| Osteoarthritis | -0.0106 | 0.0186 | -0.57 |
| Back pain | -0.2732 | 0.0235 | -11.61 |
| Cancer | 0.0539 | 0.0140 | 3.86 |
| COPD | 0.1333 | 0.0148 | 8.99 |
| Dementia | -0.2879 | 0.0342 | -8.42 |
| Schizophrenia | 0.3146 | 0.0599 | 5.25 |
| Depression | -0.0754 | 0.0130 | -5.81 |
| Age:Occupation Employed | -0.0066 | 0.0018 | -3.69 |
| Age:Occupation Early retirement pension | -0.0053 | 0.0023 | -2.35 |
| Age:Occupation Missing | 0.0287 | 0.0259 | 1.11 |
| Age:Occupation Other | -0.0039 | 0.0044 | -0.88 |
| Age:Occupation Sick leave, etc. | 0.0009 | 0.0032 | 0.27 |
| Age:Occupation Student | 0.0035 | 0.0105 | 0.34 |
| Age:Occupation Unemployed | -0.0091 | 0.0051 | -1.80 |
| Education Short:Calendar time | -0.0033 | 0.0017 | -1.91 |
| Education Medium:Calendar time | -0.0073 | 0.0032 | -2.30 |
| Education Long:Calendar time | -0.0093 | 0.0038 | -2.41 |
| Education Missing:Calendar time | -0.0141 | 0.0047 | -3.03 |
| Education Missing pre 1920:Calendar time | -0.0252 | 0.0036 | -6.93 |
| Calendar time:Occupation Employed | 0.0017 | 0.0021 | 0.84 |
| Calendar time:Occupation Early retirement pension | 0.0008 | 0.0024 | 0.32 |
| Calendar time:Occupation Missing | 0.0150 | 0.0496 | 0.30 |
| Calendar time:Occupation Other | 0.0192 | 0.0062 | 3.11 |
| Calendar time:Occupation Sick leave, etc. | -0.0142 | 0.0044 | -3.19 |
| Calendar time:Occupation Student | 0.0477 | 0.0264 | 1.81 |
| Calendar time:Occupation Unemployed | 0.0018 | 0.0064 | 0.28 |
| Osteoporosis:Osteoarthritis | 0.0982 | 0.0355 | 2.76 |
| Osteoporosis:COPD | 0.2911 | 0.0299 | 9.74 |
| Back pain:Dementia | 0.2321 | 0.0813 | 2.86 |
| Dementia:Schizophrenia | 0.3081 | 0.0745 | 4.14 |
| COPD:Depression | 0.1862 | 0.0256 | 7.28 |
| Osteoporosis:Back pain | 0.2069 | 0.0372 | 5.56 |
| Osteoarthritis:Back pain | 0.1758 | 0.0334 | 5.26 |
| Hypertension:High cholesterol | -0.0904 | 0.0209 | -4.32 |
| High cholesterol:Allergies | 0.1217 | 0.0183 | 6.65 |
| Stroke:Dementia | 0.4288 | 0.0551 | 7.78 |
| Hypertension:Schizophrenia | -0.2158 | 0.0653 | -3.31 |
| Sex Female:Stroke | 0.1256 | 0.0237 | 5.31 |
| Sex Female:Hypertension | 0.2130 | 0.0226 | 9.44 |
| Sex Female:High cholesterol | -0.1143 | 0.0164 | -6.97 |
| Sex Female:Osteoarthritis | 0.0900 | 0.0254 | 3.54 |
| Sex Female:COPD | -0.0550 | 0.0213 | -2.59 |
| Age:High cholesterol | -0.0249 | 0.0008 | -31.18 |
| Education Short:Back pain | 0.0460 | 0.0306 | 1.50 |
| Education Medium:Back pain | 0.0077 | 0.0590 | 0.13 |
| Education Long:Back pain | 0.1827 | 0.0726 | 2.52 |
| Education Missing:Back pain | -0.0603 | 0.0876 | -0.69 |
| Education Missing pre 1920:Back pain | -0.2846 | 0.0586 | -4.86 |
| Calendar time:High cholesterol | 0.0348 | 0.0016 | 21.23 |
| Calendar time:COPD | 0.0115 | 0.0019 | 6.14 |
