# Supplementary material for: Longitudinal models for the progression of disease portfolios in a nationwide chronic heart disease population
Source: PLoS One. 2023 Apr 20;18(4):e0284496. doi: 10.1371/journal.pone.0284496 (PMC10118194; doi:10.1371/journal.pone.0284496)
Supplement: S1 Text — (PDF) [file pone.0284496.s022.pdf]

S1 Text. Supplementary Methods

Variable selection procedure

The full saturated model, containing all (two-way) interaction terms between explanatory variables, contains 300+ parameters, a number that we chose to diminish. We developed a general model, at first excluding interactions with chronic diagnoses, to apply to all postponement times prior to further removal of insignificant terms, and similarly for transition probabilities. Subsequently, we added relevant interactions with diagnoses for each specific portfolio. In deriving the general model, we selected relevant two-way interactions among the explanatory variables based on expert knowledge, to avoid over-parameterization and potential modelling of random noise. Age and calendar time effects were modelled using squared and cubic terms. Interaction effects between squared age and sex were considered to allow for different curvatures around the mean age, capturing e.g. a potential menopausal effect. Due to the large amount of data available, even a normally negligible effect size would result in exceedingly low p-values. To avoid small effects potentially attributable to random noise in the models, we performed a comprehensive analysis towards selection of relevant interaction effects, where each portfolio was modelled separately for both postponement time and transitions from this portfolio. S5 Fig presents an overview of the steps in the analysis. We elaborate on details of each step in the following.

Firstly, a backwards selection starting from the general model was performed for each unique disease portfolio with at least 100 observations, using likelihood ratio tests with a 1% significance level. The models were subsequently further reduced, using a Bonferroni corrected test based on the number m of removed terms in the first reduction, i.e., an adjusted test level of $\frac{0.01}{m}$. The number of observations n_k was then calculated as the sum of the number of observations in portfolios where the model reduction did not remove the term k. With n the total number of observations in all portfolios, we calculated the fraction $\frac{n_k}{n}$, highlighting the relative importance of term k. Terms with relative importance below 1% were discarded.

Secondly, the effect sizes of each kept term across all disease portfolios were examined by visual inspection of funnel plots where the coefficients were plotted against their standard errors. The term was discarded if the plot indicated coefficients fluctuating on both sides of zero with no apparent pattern (e.g. no coefficients outside a 99% band around 0).

Finally, interactions to be included between the dichotomous diagnosis variables and the additional variables were determined by a hyper-analysis, in which coefficients for each kept variable were regressed on the diagnosis indicators.

$$\hat{\theta}_{ih} = \gamma_{0h} + \sum_{k=1}^{14} \gamma_{kh} x_{ik} + \epsilon_{ih} \tag{4}$$

where $\hat{\theta}_{ih}$ is the effect parameter for variable h estimated for portfolio i, and x_{ik} is a variable, indicating if diagnosis k is present in portfolio i or not. Each effect parameter was weighed by $w_{ih} = \frac{1}{V_{\hat{\theta}_{ih}}}$ in the analyses, where $V_{\hat{\theta}_{ih}}$ is the estimated variance of $\hat{\theta}_{ih}$. If γ_{kh} was statistically significant at the 1% level, indicating that diagnosis k influences coefficient $\hat{\theta}_{ih}$, it was considered if the interaction effect between the diagnosis and the variable h was clinically relevant. If so, the interaction term was added to the model (postponement time or transition probabilities).
